# Molecular network analysis of CPEB4 translational control and targeting CPEB4/β-catenin to modulate invasion and migration of nasopharyngeal carcinoma cells

**DOI:** 10.7150/ijms.103024

**Published:** 2025-02-18

**Authors:** Li Fen, Wei Yuanyuan, Chen Tian, Wang Yingying, Wang Yongping, Zhu Mingwan, Chen Fuhai, Zeng Manli, Tao Zezhang

**Affiliations:** 1 Research Institute of Otolaryngology-Head and Neck Surgery, Renmin Hospital of Wuhan University, Wuhan, Hubei Province, China.; 2 Department of Otolaryngology-Head and Neck Surgery, Renmin Hospital of Wuhan University, Wuhan, Hubei Province, China.; 3 Department of Otorhinolaryngology Head and Neck Surgery of Erzhou Central Hospital, Erzhou, Hubei Province, China.

**Keywords:** NPC, CPEB4, Translational control, β-catenin, Epithelial-mesenchymal transition

## Abstract

**Background:** CPEB4, an RNA-binding protein that regulates the translational efficiency of target genes, has been implicated in playing dual roles in tumor progression across multiple cancer types. This study aims to investigate the role of CPEB4 in head and neck squamous cell carcinoma (HNSCC), particularly focusing on nasopharyngeal carcinoma.

**Methods:** Differentially expressed proteins associated with CPEB4 were identified using iTRAQ-based proteomics. Consensus clustering stratified 495 HNSCC cases into two distinct molecular subtypes (C1 and C2). A prognostic risk model was constructed using LASSO regression. The effects of CPEB4 on cellular invasion and migration were analyzed in NPC cells. Western blotting assessed PI3K/AKT and WNT/β-catenin pathways and EMT markers. β-catenin nuclear activity was analyzed by immunofluorescence and western blot. A nude mouse xenograft model validated the CPEB4/β-catenin axis.

**Results:** A total of 731 proteins were identified as differentially expressed. The C2 molecular subtype exhibited significantly worse prognosis, higher tumor stemness indices, and greater sensitivity to six commonly used chemotherapeutic agents compared to C1. Overexpression of CPEB4 in NPC cells enhanced invasion and migration. Mechanistically, CPEB4 upregulated β-catenin expression and nuclear activity by targeting CPE elements within the CTNNB1 mRNA, which were inhibited by β-catenin inhibitors. *In vivo* experiments confirmed that CPEB4 overexpression promoted tumor invasiveness and migration, effects that were effectively suppressed by β-catenin inhibitors.

**Conclusions:** CPEB4 overexpression drives tumor progression in NPC by translationally upregulating β-catenin, thereby promoting cell invasion and migration. The poor prognosis associated with CPEB4 is linked to enhanced tumor aggressiveness. Importantly, β-catenin inhibitors effectively counteract the pro-invasive and pro-migratory effects mediated by CPEB4, underscoring their potential as therapeutic agents in CPEB4-overexpressing tumors.

## Background

Nasopharyngeal carcinoma (NPC), also known as Guangdong carcinoma, arises from the nasopharyngeal epithelium and is a common head and neck cancer in Southeast Asia 1. NPC often develops insidiously, and its anatomic location typically leads to late diagnosis and poor prognosis. Various factors, such as Epstein-Barr virus infection, genetic susceptibility, and environmental influences, contribute to NPC development. However, the exact pathogenesis of NPC remains unclear. A deeper understanding of molecular mechanisms may help identify urgently needed therapeutic targets and biomarkers.

Cytoplasmic Polyadenylation Element Binding Protein 4 (CPEB4) is an RNA-binding protein that regulates the polyadenylation and translation of specific mRNAs by binding to the cytoplasmic polyadenylation element (CPE) on their 3'UTR 2-6. CPEB4. CPEB4 plays a critical role in regulating the cell cycle, proliferation, and apoptosis 3, 7. Growing evidence highlights the role of CPEB4 in tumors 8-14. Its expression is upregulated in cancers like pancreatic cancer 14, and melanoma 12, potentially promoting tumor growth and metastasis by regulating oncogene translation. Studies have shown that knocking down CPEB4 in HepG2 hepatocellular carcinoma cells promotes colony formation *in vitro*. In an *in vivo* xenograft mouse model, CPEB4 knockdown accelerates tumor growth 15. These findings highlight the dual role of CPEB4 in tumor progression, varying across different cancer types.

Investigating CPEB4's molecular mechanism can uncover its role in pathological processes, potentially identifying new targets for diagnosing and treating NPC. This study aims to identify proteins regulated by CPEB4 in NPC cells and to analyze its associated molecular network. Using HNSCC data from the TCGA database, we will also examine the CPEB4-related gene signature, molecular subtypes, prognosis, chemoresistance, and other aspects of NPC. Furthermore, we will investigate the role and mechanisms by which CPEB4 induces the invasion and migration of NPC at the cellular level.

## Methods

### Cell culture and transfection

Normal nasopharyngeal epithelial cells NP460 and the NPC cell lines CNE2Z and 5-8F were generously provided by the laboratory of Guangxi Medical University. The cells were cultured in RPMI-1640 medium (Invitrogen Life Technologies, Carlsbad, CA, USA) supplemented with 10% fetal bovine serum (Invitrogen Life Technologies, Carlsbad, CA, USA), 20mg/ml ampicillin, and 20mg/ml kanamycin. All cells were maintained at 37°C in a humidified incubator with 5% CO_2_.

NPC cells (2×10^5^ cells/well) were transfected with CPEB4 overexpression or RNAi lentiviral vector constructs (MOI=20, provided by GeneChem Co., Ltd, Shanghai, China). Stable transfected CNE-2Z and 5-8F cell lines were selected by culturing them for 1 week in complete medium (RPMI1640 with 10% FBS, ampicillin and kanamycin), supplemented with puromycin (2 µg/ml).

### Isobaric Tags for Relative and Absolute Quantitation (iTRAQ)

Samples were prepared using SDT lysis, and protein quantification was performed using the BCA method. The samples were aliquoted and stored at -80°C. For each sample, 20 µg of protein was loaded onto a 12% SDS-PAGE gel and stained with Coomassie Brilliant Blue. After FASP digestion, 100 µg of peptide from each sample was labeled according to the instructions of the iTRAQ Labelling Kit (AB SCIEX). Labeled peptides from each group were pooled and fractionated using the Agilent 1260 Infinity II HPLC system. The samples were lyophilized and reconstituted in 0.1% formic acid, then divided into portions and separated using an Easy nLC system with nanoliter flow rates. The samples were separated by chromatography and analyzed using a Q-Exactive mass spectrometer. The raw mass spectrometry data were identified and quantified using Mascot 2.5 and Proteome Discoverer 2.1.

### Bioinformatics analysis

HNSCC data were downloaded from the TCGA database, and a CPEB4-based gene signature network analysis was performed using iTRAQ-screened 731 differentially expressed proteins (CPEB4-DEPs) (Figure A: Flowchart). The analysis was conducted in R (v4.0.3), and correlation was evaluated using Spearman correlation analysis.

Consensus clustering analysis was conducted using the R package ConsensusClusterPlus (v1.54.0). A total of 495 HNSCC samples were classified into C1 and C2 phenotypes. Kaplan-Meier survival curves were generated using the survival and survminer packages.

#### Tumor stemness index

The OCLR algorithm was used to calculate the mRNAsi, as constructed by Malta *et al.* Based on the mRNA expression signature, the gene expression profile contained 11,774 genes. The minimum value was subtracted, and the result was normalized by dividing by the maximum, mapping the tumor stemness index to the range [0,1]. Differences in the tumor stemness index between subtypes C1 and C2 were compared.

IC50 prediction for chemotherapeutic response was conducted for each sample using the largest publicly available pharmacogenomics database [the Genomics of Drug Sensitivity in Cancer (GDSC), https://www.cancerrxgene.org/]. The prediction was implemented with the R package “pRRophetic.” Ridge regression was used to estimate the samples' half-maximal inhibitory concentration (IC50). Six commonly used chemotherapeutic agents for head and neck squamous carcinoma—cisplatin, 5-fluorouracil, gemcitabine, paclitaxel, docetaxel, and methotrexate—were selected to compare the IC50 differences between subtypes C1 and C2.

#### Least Absolute Shrinkage and Selection Operator Regression Analysis (Lasso)

Data in TPM format were extracted and normalized to log2(TPM+1). A total of 495 samples were used to construct prognostic prediction models. CPEB4-PRGs were utilized to identify prognostic-related risk factors. Independent variables were screened using Lasso regression, where regression coefficients were penalized by the L1 norm. This approach compressed the coefficients of unimportant variables to 0, enabling variable screening and dimensionality reduction. Screened variables were used as independent variables, and survival time was set as the dependent variable. A multifactorial COX regression model was constructed based on the λ value (lambda.min or lambda.1se) obtained from Lasso analysis. The risk score was then calculated, and a risk score model was constructed. Survival analyses were conducted using Kaplan-Meier curves to compare survival differences between high-risk and low-risk groups. ROC curves were plotted to determine model thresholds and measure its accuracy. ROC curves for three time points (1, 3, and 5 years) were plotted using the timeROC package, and AUC values were calculated.

#### Enrichment analysis of GO annotations and KEGG annotations

The significance of protein enrichment for a given GO term or KEGG pathway was assessed using Fisher's Exact Test by comparing the distribution of individual GO classifications or KEGG pathways between the target protein set and the overall protein set.

The URLs of web tools used for other bioinformatics analyses in this study are as follows: (1) scRNA sequencing analysis of HNSCC: TISCH2 (http://tisch.comp-genomics.org/home/); (2) Prediction of phase-separated molecular structures: PLAAC (http://plaac.wi.mit.edu/); (3) Structure prediction of non-classical secretory proteins: TMHMM (https://services.healthtech.dtu.dk/services/TMHMM-2.0/); and (4) Signal peptide prediction: SignalP (https://services.healthtech.dtu.dk/services/SignalP-6.0/).

#### Combinatorial code for CPE-mediated translational control

First, the 3'UTR base sequence of the target gene mRNA was obtained using the R package 'biomaRt'. The R version used was 4.3.2. The combination characteristics of base sequences related to CPE elements, as reported in the literature 16, were used for comparison. The sequence includes the following features:

Hexa (Hexanucleotide): AATAAA, ATTAAA, (A[AT]TAAA)

CPEC (Consensus CPE): TTTTAT, TTTTAAT, (TTTTAA?T)

The CPE Combined Code Identification web tool (https://genome.crg.eu//CPE/server.html), as described in this literature, was used to analyze the results, and the Prosite tool was utilized to plot the patterns.

### Western blotting

The cells were harvested and lysed in a buffer containing protease and phosphatase inhibitors (Roche, Switzerland). The lysates were resolved by SDS-PAGE, transferred to PVDF membranes (Merck Millipore, USA), and immunoblotted with specific primary antibodies. After immunoblotting with a goat anti-rabbit secondary antibody (Biosharp, China), the membranes were scanned using the ChemiDoc chemiluminescence imaging system (Bio-Rad, USA).

### Colony formation assay

After digestion, cells in the logarithmic growth phase were resuspended into a cell suspension and counted. Inoculate 1,000 cells per well for each experimental group in 6-well culture plates. Cells were continuously cultivated for 14 days. Fix the cells with 4% formalin for 30 minutes, followed by staining with crystal violet for 15 minutes. After washing carefully with PBS, allow the cells to dry. Positive clones (each containing >50 cells) were observed under a microscope. The number of clones and the cloning efficiency were then calculated.

### Wound healing assay

The cell concentration was adjusted to 5 × 10⁵ cells/ml, and 1 ml of the cell suspension was inoculated into a six-well plate. After the cells attached, the serum-free medium was replaced and incubated for 12 hours. A pipette tip was used to draw a line perpendicular to the well plate on the cell surface, creating cell scratches. PBS was used to wash and remove the scratched cells. Photographs were taken at different times (0, 24, and 48 hours) under a microscope. Scratch widths were analyzed and calculated with ImageJ software.

### Transwell invasion assay

The Matrigel working solution was prepared with serum-free medium at an 8:1 ratio with Matrigel gel (BD Biosciences, San Jose, CA, USA). 80 μl of the working solution was added to the upper chamber of a Transwell and incubated at 37°C for 2 hours to allow the Matrigel to gel. The cells were digested, followed by centrifugation and collection in the blotting chamber. The cells were digested, centrifuged, and then collected in sterile centrifuge tubes. The cell concentration was adjusted to 5 × 10^5^ cells/ml. Subsequently, 100 μl of the cells was inoculated into the upper chamber, and 600 μl of the medium with 20% FBS was added to the lower chamber. After 24 hours of incubation, the cells in the upper chamber were removed with a medical swab. The cells were fixed in 10% formaldehyde for 30 minutes, rinsed with PBS, stained with 0.1% crystal violet for 10 minutes, and then the chambers were air-dried after being removed from the PBS rinses. The cells were photographed using an Olympus microscope for further analysis.

### RNA immunoprecipitation assay

The EZ-Magna RIP kit (from Millipore, Billerica, MA, USA) was utilized to conduct the RIP assay. A total of 1.0 × 10⁷ CNE-2 cells were collected and lysed in the RIP lysis buffer. Subsequently, protein A/G magnetic beads were incubated with either the IgG antibody (from Millipore, Billerica, MA, USA) or the CPEB4 antibody (ab155204, from Abcam, Cambridge, MA, USA). The RNA was eluted from the magnetic bead-binding complexes following the manufacturer's instructions. Finally, an agarose PCR assay was carried out to detect the β-catenin mRNA using antibodies against IgG or CPEB4.

### Nude mice transplant tumor model

Fifteen male nude mice, aged 3 - 4 weeks, were procured from Vital River Laboratory Animal Technology (Beijing, China) and reared under specific pathogen-free conditions. The animal experiments were authorized by the Medical Ethics Committee of Renmin Hospital of Wuhan University. After a one-week adaptive feeding period, the nude mice were randomly and evenly allocated to the control group, the CPEB4 overexpression group, and the CPEB4 overexpression + MSAB group. Suspend the cells in serum-free medium at a concentration of 1 × 10⁷/ml. 0.1 ml of the cell suspension was subcutaneously injected into the right axilla of each nude mouse.

One week after cell inoculation, the mice were treated via intraperitoneal injection with vehicle or MSAB (at a dose of 20 mg/kg) daily for two weeks. Two weeks after the intraperitoneal injection, the mice were euthanized and the tumor weight was measured. The tumor dimensions were measured, and the volume was calculated based on the length (L) and width (W) using the formula (volume = (L × W²)/2).

### Immunohistochemical staining (IHC)

The two-step method was employed for the immunohistochemical analysis. The slides were dried, dewaxed, and subsequently rehydrated in gradient ethanol concentrations. After pretreatment in citrate buffer for antigen retrieval, the slides were then immersed in 0.3% hydrogen peroxide to block endogenous peroxidase activity. Subsequently, the slides were incubated with the primary antibody (at a dilution of 1:200; Cell Signaling Technology) overnight at 4°C. They were then rinsed with PBS for 5 minutes and incubated with the secondary antibody (Poly-HRP Goat anti-rabbit; Maixin; Bio, Fuzhou, China) for 30 minutes at room temperature. The slides were stained with 3, 3-diaminobenzidine (DBA) for 5 minutes, counterstained with Mayer's hematoxylin for the cell nucleus, dehydrated, and mounted.

### Statistical analysis

All experiments were performed in triplicate. Results are expressed as means ± standard deviations of three independent experiments. P-value calculations were conducted using unpaired Student's T-tests for two-group comparisons. Kaplan-Meier (KM) survival curves were compared between different groups using the Log-rank test. The significance between the two groups for Tumor stemness index and IC50 were assessed using the Wilcoxon rank-sum test. P < 0.05 was considered statistically significant.

## Results

### CPEB4 promotes proliferation, invasion, and migration of NPC cell lines

**Figure 1:**
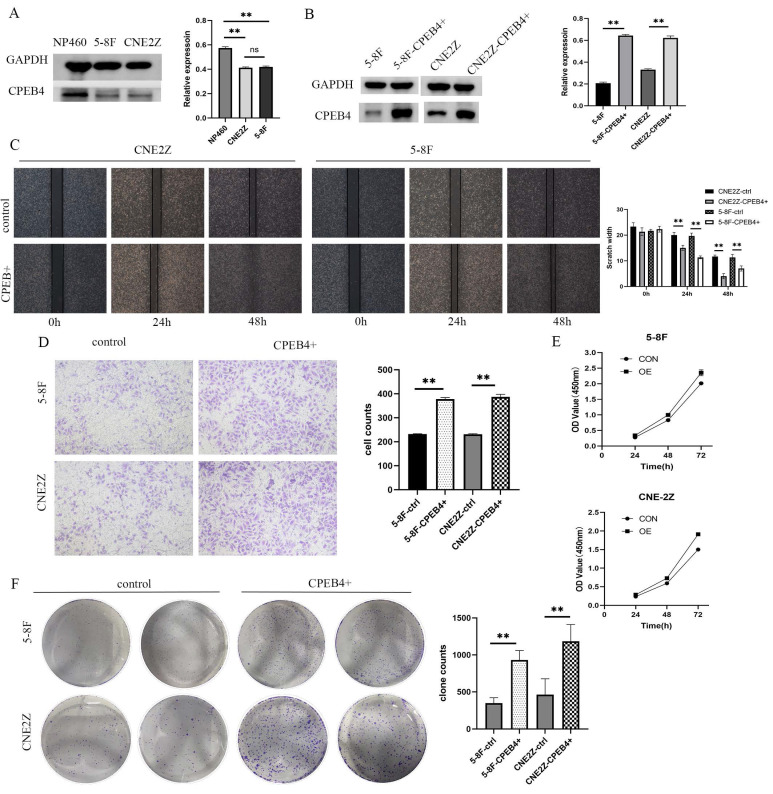
Expression of CPEB4 in NPC cells and its role in promoting cancer phenotypes. (A) Western blot analysis revealed that CPEB4 expression was lower in NPC cell lines (5-8F, CNE2Z) compared to normal nasopharyngeal epithelial cells (NP460). (B) Lentiviral overexpression of CPEB4 was successfully established in NPC cell lines 5-8F and CNE2Z. (C) Scratch assay showed that overexpression of CPEB4 enhanced NPC cell migration. (D) Transwell assay demonstrated increased invasion ability of NPC cells after CPEB4 overexpression. (E) CCK-8 assay indicated that CPEB4 overexpression enhanced the proliferation of NPC cells. (F) Colony formation assay confirmed that CPEB4 overexpression promoted colony-forming ability.

**Figure 2:**
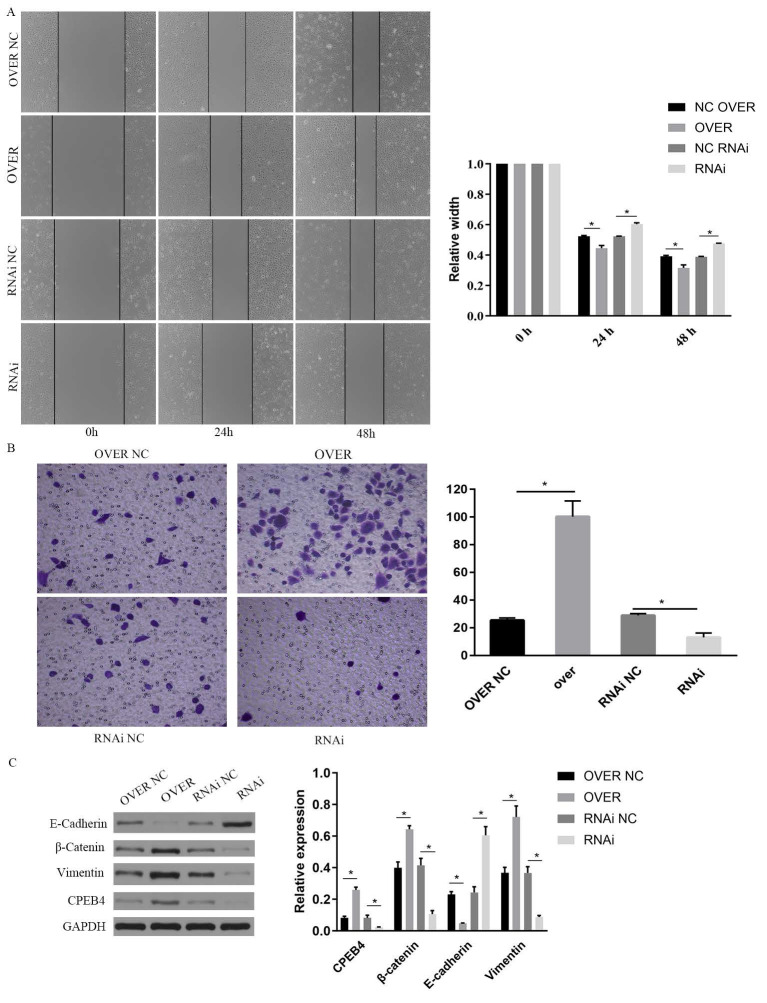
Impact of CPEB4 overexpression and knockdown on NPC cell migration and invasion. (A) Scratch assay demonstrated that CPEB4 overexpression enhanced migration ability, whereas RNAi knockdown reduced it. (B) Transwell assay showed that invasion ability increased with CPEB4 overexpression and decreased after RNAi knockdown. (C) Western blot analysis of EMT markers (E-cadherin, vimentin, and β-catenin) revealed that CPEB4 overexpression increased β-catenin and promoted EMT, while knockdown reversed these effects.

To investigate the role of CPEB4 in NPC cells and the underlying mechanisms, two NPC cell lines (5-8F/CNE2Z) and a normal nasopharyngeal epithelial cell line (NP460) were selected, and the expression of CPEB4 was examined. We found that the expression of CPEB4 was lower in cancer cells (Figure 1A), consistent with the results in HNSCC (Figure S1) and NPC tumor tissue (as reported in our previous research). Subsequently, lentiviral vectors were used to construct stably transfected NPC cell lines with CPEB4-targeted overexpression and interference (5-8F-CPEB4+; CNE2Z-CPEB4+) (Figure 1B) and (CNE2Z OVER/RNAi) (Figure 2C). The transfected cell lines were assessed for proliferation, apoptosis, invasion, and migration. The results (Figure 1C - F, Figure 2A/B) indicated that NPC cells displayed an enhanced malignancy phenotype, including accelerated proliferation, decreased apoptosis, increased clonal formation, and enhanced invasion/migration after CPEB4 overexpression. The expression characteristics of CPEB4 at the tissue and cellular levels seem to be contradictory. To account for this phenomenon, we analyzed the molecular characteristics of CPEB4 (Figure S2/S3). Firstly, CPEB4 may be a non-classical secreted protein. It lacks a transmembrane structure and a signaling peptide, and has a secP parity score of 0.91 (>0.6). Secondly, by using single-cell sequencing data to analyze the cellular composition of HNSCC, it was discovered that CPEB4 was highly expressed in immunophagocytes like plasma cells, mast cells, and macrophages. It appears that CPEB4, with its dual nature, plays a crucial role in the developmental process. However, it might be secreted to the immune phagocytes in the microenvironment and play a pro-cancer role during tumor formation. Nevertheless, the precise mechanism requires further investigation.

### iTRAQ screening identifies CPEB4-associated differential proteins, followed by cluster analysis and molecular subtyping with prognostic implications

Using iTRAQ, 731 differentially expressed proteins (CPEB4-DEPs) were screened. Subsequently, GO and KEGG enrichment analyses were conducted. The results (Figure S5) indicated that the GO enrichment was in aspects such as metabolism, stress, signaling pathways, migration, and DNA junctions, while the KEGG enrichment was in pathways including cell adhesion, autophagy, HPV, EBV, endocytosis, and metabolism.

**Figure 3:**
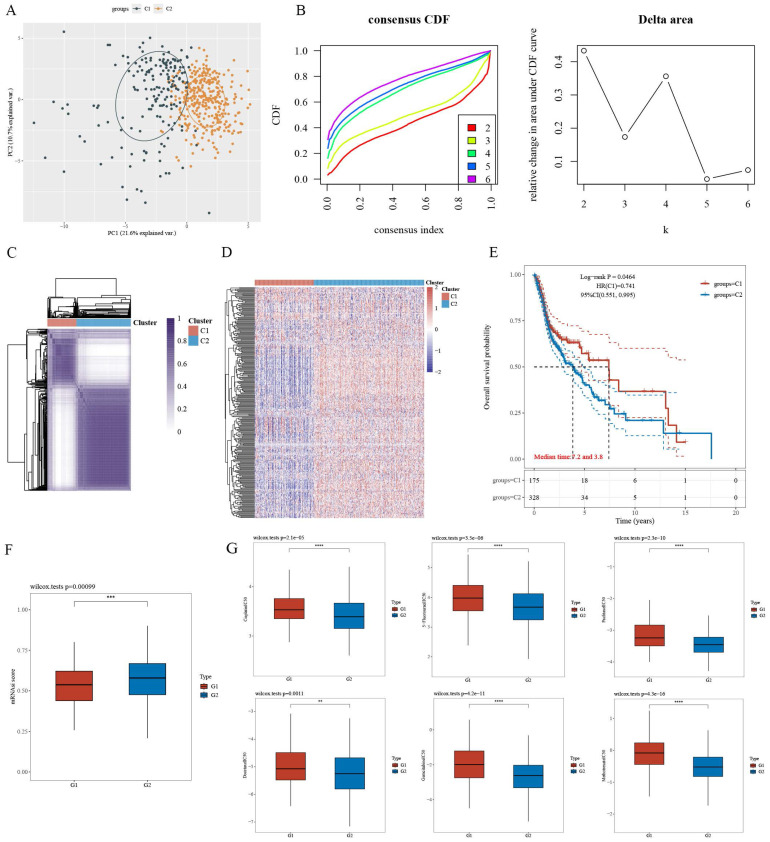
Differentially Expressed Proteins (DEPs) related to CPEB4 and molecular subtyping of HNSCC. (A) PCA revealed clear separation between the two molecular subtypes (C1 and C2). (B) Consensus clustering identified the optimal k=2, with the consensus index and delta area plotted. (C) Consensus matrix for k=2 clusters. (D) Heatmap showed that subtype C2 had high expression of differential genes. (E) Prognosis analysis revealed that subtype C2 had significantly worse survival outcomes compared to C1. (F) Tumor stemness index was significantly higher in subtype C2. (G) IC50 analysis of six chemotherapeutic drugs (cisplatin, 5-fluorouracil, paclitaxel, gemcitabine, methotrexate, and docetaxel) indicated that subtype C2 was more sensitive (lower IC50 values) than C1.

For CPEB4-DEGs, the differentially expressed genes (DEGs) in the 731 CPEB4-DEPs within TCGA-HNSCC were screened, and 279 CPEB4-DEGs were obtained. These CPEB4-DEGs were then utilized as the gene signature for the molecular classification of HNSCC. Specifically, 495 cases of HNSCC with complete metadata in the TCGA database were classified into two subtypes (C1 and C2) through unsupervised consensus clustering analysis (Figure 3A - D). Subsequently, the Kaplan-Meier curve was plotted (Figure 3E), revealing that C2 (the group with high expression of differential genes) had a poorer prognosis than C1, with median survivals of 3.8 and 7.2 years respectively.

A differential analysis of the tumor stemness index for C1 and C2 (Figure 3F) was performed. The results suggested that the tumor stemness index of C2 was significantly higher than that of C1, which was consistent with the outcomes of the prognostic analysis.

A differential analysis of the IC50 values of commonly used chemotherapeutic drugs for HNSCC (including cisplatin, 5-fluorouracil, paclitaxel, docetaxel, methotrexate, and gemcitabine) was carried out for the C1 and C2 subtypes (Figure 3G). The results indicated that the IC50 of C2 was significantly lower than that of C1, suggesting that targeting CPEB4 might have a synergistic effect on chemotherapy.

### Construction of a LASSO prognostic model using iTRAQ-identified CPEB4 target proteins and prognostic analysis

**Figure 4:**
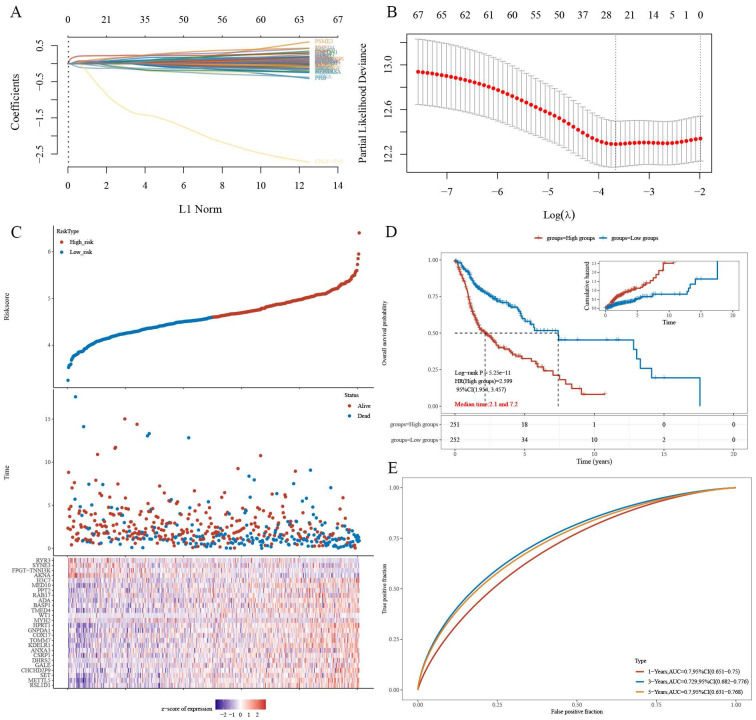
Prognostic model for HNSCC based on CPEB4-DEPs. (A) Regression coefficients for risk genes were determined using LASSO analysis. (B) LASSO curve analysis identified the λ values for lambda.min and lambda.1se. (C) Risk score model was constructed based on individual risk scores. (D) Kaplan-Meier curves showed significant differences in survival between high- and low-risk groups. (E) ROC curves demonstrated the model's predictive performance with AUC values of 0.7, 0.729, and 0.7 for 1, 3, and 5 years, respectively.

The gene set CPEB4-PRGs (118) was screened first: TCGA-HNSCC prognosis-related genes (PRGs) in 731 CPEB4-DEPs (Figure A: Flowchart); (Figure 4A) each curve in the graph represents one gene; (Figure 4B) the graph of each vertical line represents one gene, and the two dashed lines are the results of lambda.min or lambda.1se, respectively. lambda.min=0.0257; Riskscore was calculated and the risk score model was constructed (Figure 4C/D). Riskscore was calculated as follows:

Riskscore=(0.065)*RSL1D1+(0.0374)*METTL5+(0.0014)*SET+(0.0709)*CHCHD2P9+(0.0164)*GALE+(0.0106)*DHRS2+(0.113)*CSRP1+(0.0499)*ANXA3+ (0.0036)*KDELR1+(0.0355)*TOMM7+(0.0273)*COX17+(0.0299)*GNPDA1+(0.228)*HPRT1+(0.0349)*MYH2+(0.021)*WT1+(0.1644)*TMED4+(0.0725)* BASP1+(0.0171)*ADA+(0.0403)*RAB17+(0.0138)*PPT2+(-0.0774)*MED10+(0.1752)*H3C7+(-0.1416)*AKNA+(-1.2139)*FPGT-TNNI3K+(-0.1084)*SYNE3 +(-0.1125)*RYR3

There was a significant survival difference between the high-risk group and the low-risk group (Figure 4D), with a median survival time of 2.1/7.2 years for the two groups, respectively. ROC curves were plotted for three time points (1/3/5 years), and AUC values for three time points (1/3/5 years) were 0.7/0.729/0.7, respectively (Figure 4E). The AUC values indicate that this risk-prognostic model has high classification performance.

### CPEB4 translational regulation activates Wnt pathway and promotes EMT

To investigate the role of CPEB4 in NPC cells and its underlying mechanisms, we overexpressed CPEB4 in NPC cells and observed enhanced cell invasion and migration. We employed western blot to assess the key signaling pathways involved in invasion and migration, namely the Wnt and PI3K/AKT pathways, and found that key proteins including β-catenin, p-AKT, and p-GSK3β were significantly upregulated following CPEB4 overexpression (Figure 6D). Concurrently, the expression of mesenchymal markers vimentin and N-cadherin increased, while E-cadherin expression decreased, suggesting that CPEB4 may promote EMT in NPC cells (Figures 2C and 6D).

**Figure 5:**
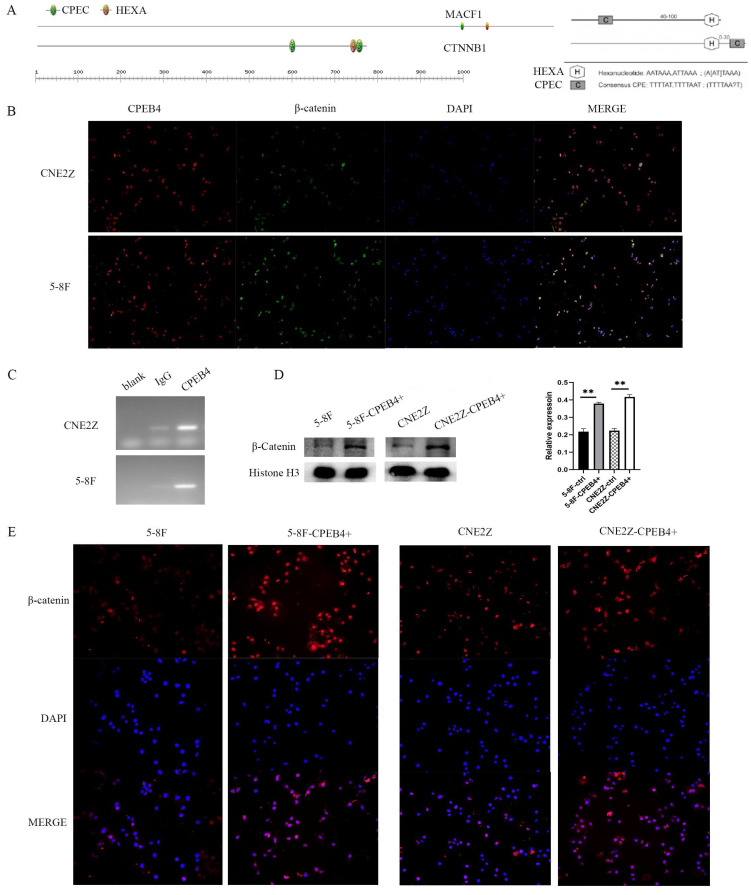
CPEB4 regulates β-catenin translation. (A) Analysis of the 3'UTR region of CTNNB1 revealed CPE elements essential for translational activation by CPEB4. (B) Immunofluorescence showed colocalization of CPEB4 and β-catenin in the cytoplasm and nucleus. (C) RIP assay confirmed that CPEB4 binds to CTNNB1 mRNA in NPC cell lines (CNE2Z and 5-8F). (D) Western blot revealed increased nuclear β-catenin levels after CPEB4 overexpression. (E) Immunofluorescence showed elevated nuclear β-catenin levels in NPC cells with CPEB4 overexpression.

Screening for genes significantly associated with CPEB4 expression (correlation coefficient>0.5) in the TCGA HNSCC data yielded 913 related genes; cross-matching with CPEB4-DEPs identified 44 CPEB4-related genes (CPEB4-CRGs). Among them, MACF1, with a correlation coefficient of 0.629, was analyzed for its mRNA 3'UTR region and found to contain CPEB4 activation-associated CPE elements (Figure 5A). A literature review revealed that MACF1 significantly induced PI3K/AKT and GSK3β activities and was closely associated with tumor metastasis. Structural analysis revealed SH3 domains and a disordered structure in MACF1, and MACF1 isoform2 was reported to form complexes with CTNNB1 and GSK3B (UniProt), suggesting that MACF1 may regulate phosphorylation activity by directly binding to GSK3β. In our study, following CPEB4 overexpression, p-AKT and p-GSK3β expressions were significantly increased, presumably due to the translational regulation of MACF1 by CPEB4.

Concurrently, we found that β-catenin, a key Wnt pathway protein with elevated expression following CPEB4 overexpression, is also among the 731 CPEB4 DEPs. We analyzed the 3'UTR region of the encoding gene, CTNNB1 mRNA, finding CPE activation-associated elements (Figure 5A) (CPEC (TTTTAT/TTTTAAT) located 46-100 nt (70 nt) before HEXA (ATTAAA) or 0-30 nt (1 nt) after HEXA (ATTAAA)). As activated β-catenin enters the nucleus to promote EMT-associated molecule expression, we hypothesized that CPEB4 might promote EMT by targeting the translational regulation of β-catenin expression, thereby increasing β-catenin activity and promoting its nuclear entry, which would increase the expression of EMT-related mesenchymal molecules.

### CPEB4 upregulates β-catenin expression and activity via translational regulation of CTNNB1 mRNA

After identifying that the 3'UTR region of CTNNB1 mRNA contains CPEB4 activation-associated CPE elements, we conducted a RIP assay (Figure 5C) using magnetic beads with adsorbed CPEB4 antibody in CNE2Z and 5-8F cells. RT-PCR was performed on the bound RNA, and agarose gel electrophoresis revealed CTNNB1 bands. These RIP results indicate that CPEB4 binds to CTNNB1 mRNA, potentially regulating β-catenin expression through translational control.

To further investigate the impact of CPEB4 overexpression on β-catenin activity, we assessed nuclear β-catenin expression using immunofluorescence (Figure 5E). The results indicated a significant increase in nuclear β-catenin expression following CPEB4 overexpression (Figure 5E). This increase was further confirmed by western blot (Figure 5D), suggesting that enhanced β-catenin entry into the nucleus after CPEB4 overexpression activates the EMT molecular pathway, thereby promoting the invasion and migration of NPC cells.

### β-catenin inhibitors suppress CPEB4 overexpression-induced invasion and migration in NPC cells

The intervention and validation of the roles of CPEB4 and its effector molecule β-catenin were conducted. We found that CPEB4 overexpression enhanced invasiveness and migration and stimulated β-catenin expression, while β-catenin inhibitors mitigated the increase in invasiveness and migration caused by CPEB4 overexpression.

**Figure 6:**
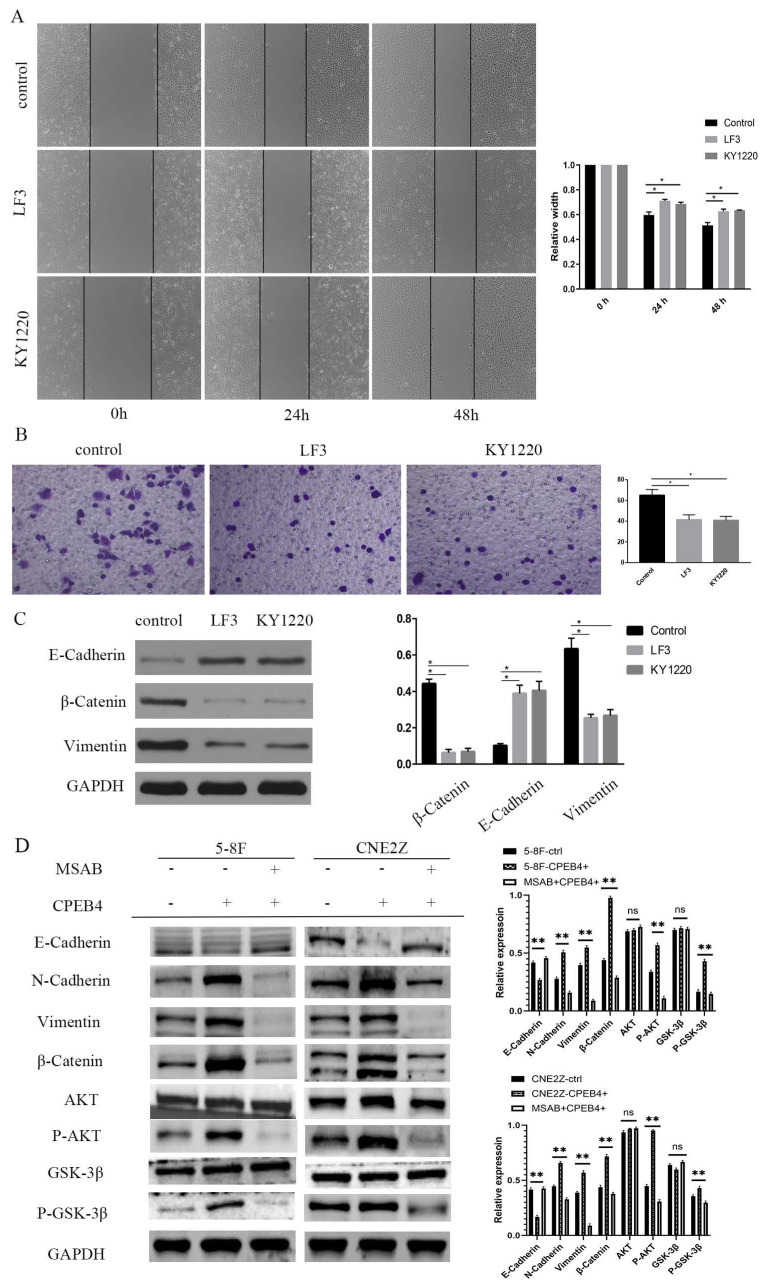
Effect of β-catenin inhibitors on CPEB4-induced invasion and migration. (A) Migration ability induced by CPEB4 overexpression was significantly reduced by β-catenin inhibitors LF3 and KY1220. (B) Invasion ability was similarly suppressed by LF3 and KY1220. (C) Western blot analysis showed that LF3 and KY1220 inhibited mesenchymal markers (vimentin, β-catenin). (D) Overexpression of CPEB4 increased mesenchymal markers (vimentin, β-catenin, p-AKT, p-GSK3β), which were suppressed by the β-catenin inhibitor MSAB.

CNE2Z cells with CPEB4 overexpression and knockdown were treated with β-catenin inhibitors LF3 and KY1220 (20 μmol/L for 2 days), and 5-8F-CPEB4+ and CNE2Z-CPEB4+ cells were treated with MSAB (5 μmol/L for 24 h), respectively. Scratch assay results indicated that the scratch width in the LF3 and KY1220 groups was significantly narrower than that in the control group at 48 hours (Figure 6A), and the number of cells penetrating the membrane in the LF3 and KY1220 groups was significantly lower than in the control group in the transwell assay (Figure 6B). Western blot assays detected changes in the expression of EMT molecules, the PI3K/AKT pathway, the Wnt pathway, and other molecules. E-cadherin expression increased, while β-catenin and vimentin expression decreased, and the expression of p-AKT and p-GSK3β also decreased (Figure 6C/D), suggesting that the invasion and migration induced by CPEB4 overexpression were significantly inhibited.

### β-catenin inhibitor MSAB reduces CPEB4 overexpression-induced EMT *in vivo*

**Figure 7:**
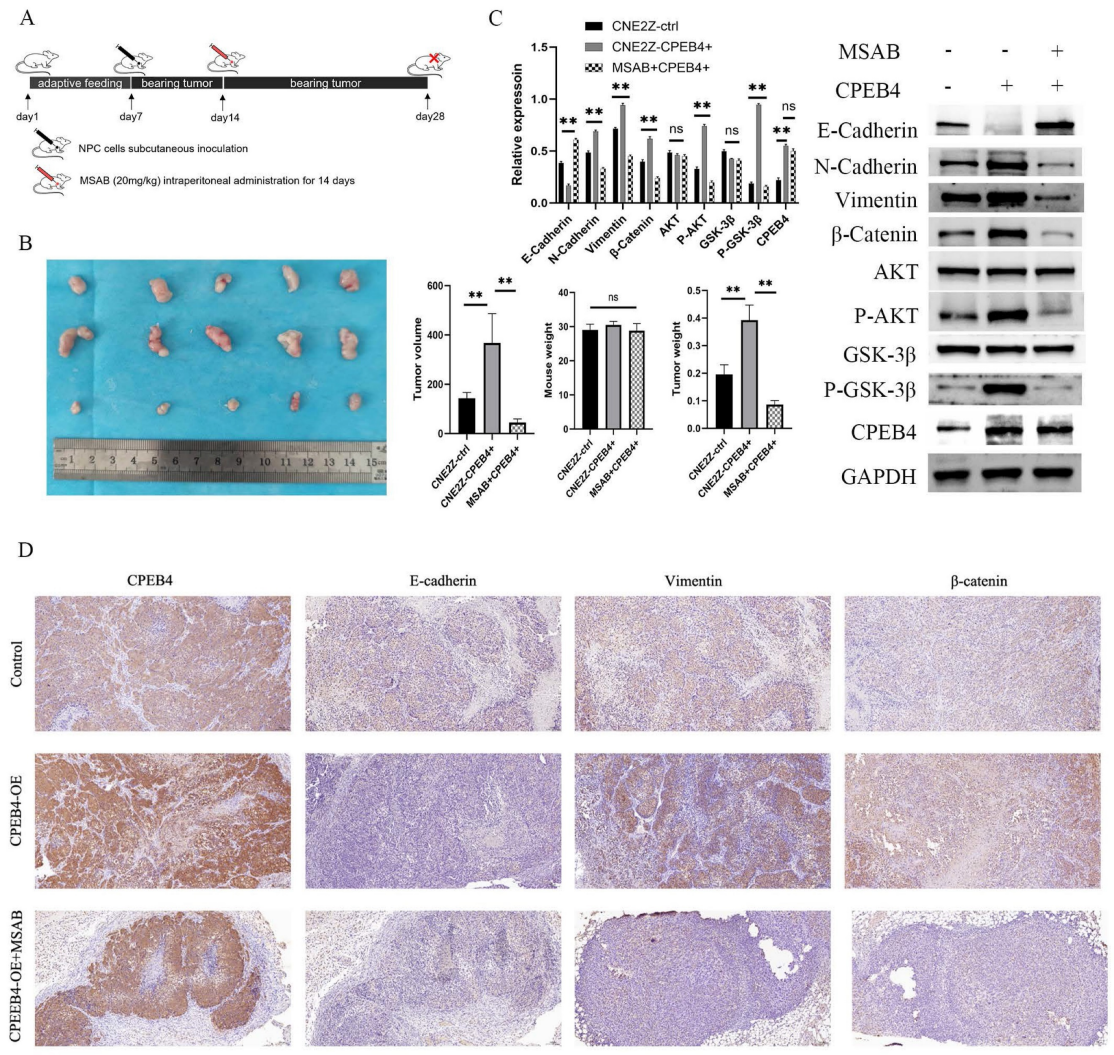
*In vivo* validation of the CPEB4/β-catenin axis in NPC EMT. (A) Nude mice were subcutaneously inoculated with CNE2Z cells (control or CPEB4 overexpression) with or without MSAB (20 mg/kg for 14 days). (B) Tumor growth and weight curves showed significant reduction in tumor size in the MSAB-treated group. (C) Western blot analysis revealed that CPEB4 overexpression increased mesenchymal markers, p-AKT, p-GSK3β, and β-catenin, which were decreased in the MSAB-treated group. (D) IHC analysis showed decreased E-cadherin and increased vimentin/β-catenin in the CPEB4 overexpression group, with opposite trends observed in the MSAB group.

To verify the effect of CPEB4 on the expression of CTNNB1 *in vivo*, we employed a nude mouse xenograft tumor model (Figure 7A). After a 7-day acclimatization period, nude mice were dorsally subcutaneously inoculated with CPEB4-overexpressing CNE2Z cells or control CNE2Z cells. One group of the CPEB4-overexpressing group received intraperitoneal injections of MSAB treatment (20 mg/kg) for 14 days. On day 28, the mice were sacrificed, and the tumors were excised and measured (Figure 7B). Tumor volume was significantly larger in the CPEB4-overexpressing group and was substantially reduced following MSAB intervention. Tumor tissue proteins were extracted for western blot analysis (Figure 7C) and IHC staining (Figure 7D). Western blot results indicated that MSAB could inhibit the overexpression-induced increase in p-AKT, p-GSK3β, and mesenchymal markers Vimentin and β-catenin. IHC detection of the epithelial marker E-cadherin and mesenchymal markers Vimentin and β-catenin yielded similar findings.

## Discussion

CPEB4 exhibits dual roles in various tumors, functioning as an oncogene in certain cases like hepatocellular carcinoma (HCC) and as a pro-carcinogen in others such as pancreatic ductal adenocarcinoma and glioblastoma.

Our previous study revealed that CPEB4 is downregulated in HNSCC, based on GEO database analysis and immunohistochemistry of FFEP tissues, suggesting its potential role as a tumor suppressor in HNSCC 17. In this study, we analyzed CPEB4 expression in a cohort of 504 HNSCC cases from the TCGA database. The findings revealed significantly lower CPEB4 expression in HNSCC tissues compared to paraneoplastic tissues, prompting further investigation into its potential tumor-suppressive role. In cellular experiments, we observed reduced CPEB4 protein levels in tumor cells compared to normal cells. Surprisingly, overexpression of CPEB4 in tumor cells resulted in enhanced malignant behaviors, including proliferation, invasion, and migration.

CPEB4 has been reported to exhibit dual roles in tumors, with its protein levels significantly upregulated in early-stage HCC but reduced in late-stage HCC, as observed in paired tumor and adjacent non-tumor liver specimens from 49 HCC patients 15. We analyzed CPEB4 expression in early versus advanced HNSCC using the TCGA database and found no significant differences (Figure S1D).

Subsequently, we reviewed extensive data to understand the contradictory findings 15, 18, focusing on factors like CPEB4's role in embryonic development and its regulation in normal and tumor tissues. One possible explanation is that CPEB4, as a secreted protein, is more abundant in mononuclear macrophages, mast cells, and plasma cells within the tumor microenvironment than in tumor cells, potentially promoting a pro-tumorigenic phenotype.

CPEB4 can form phase separation 19 (Figure S2A), a key mechanism for assembling biomolecular condensates 20-22. Phase separation regulates various cellular processes, such as gene transcription and responses to external stimuli, while its dysregulation may contribute to cancer development. Further investigation is required to determine whether CPEB4 plays distinct roles in formed versus unformed phase separation.

Our subsequent experiments, focusing on NPC cells and transplanted tumor models in nude mice, investigated how CPEB4 overexpression promotes malignant tumor phenotypes. Using the TCGA database of HNSCC clinical data, we screened CPEB4-related genes, constructed molecular subtypes, and conducted prognostic analyses to address the paradox of reduced CPEB4 expression in HNSCC tissues but pro-tumorigenic behavior after overexpression in cells.

Subsequent cellular experiments explored and validated the role of the CPEB4/β-catenin axis in regulating EMT in NPC cells. Phenotypic studies showed that overexpression of CPEB4 enhanced the invasive and migratory abilities of NPC cells, while β-catenin inhibitors suppressed these effects. Molecular and signaling pathway studies revealed that CPEB4 overexpression significantly induced the expression of EMT mesenchymal molecule N-cadherin, as well as activation of the Wnt, PI3K/AKT pathway, p-AKT, p-GSK3β, and β-catenin. These effects were suppressed by β-catenin inhibitors. Structural and interaction analyses showed that the mRNA 3'UTR region of CTNNB1, which encodes β-catenin, contains CPE activation-related components. RIP experiments confirmed that CPEB4 can bind to CTNNB1 mRNA. Nuclear analysis revealed that β-catenin expression and intranuclear translocation increased after CPEB4 overexpression, indicating that CPEB4 activates β-catenin and promotes EMT. *In vivo* experiments demonstrated that CPEB4 overexpression promotes tumor growth, while β-catenin inhibitors reduce tumor size in mice. β-catenin inhibitors effectively block CPEB4's tumor-promoting effects, making them potential targets for therapeutic intervention.

One limitation of this study is the lack of experimental validation to confirm whether CPEB4 is secreted by other cells in the microenvironment and influences tumor cells. Another limitation is that the iTRAQ screening technique identified downstream proteins with altered expression but did not investigate CPEB4's role as an RNA-binding protein acting on CPE elements. Combining these methods may yield more comprehensive experimental results. This study may not provide a complete chain of evidence demonstrating the pro-tumorigenic role of CPEB4 in HNSCC. However, our analysis of its seemingly contradictory roles suggests possible explanations and offers ideas and solutions for reference.

## Supplementary Material

Supplementary materials and methods, figures.

## Figures and Tables

**Figure A:**
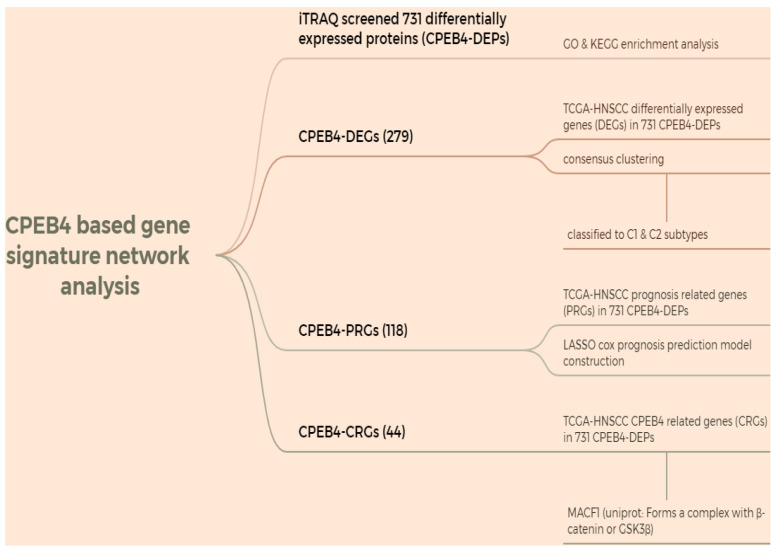
Flowchart.

## Abbreviations

HNSCC: head and neck squamous cell carcinoma
NPC: Nasopharyngeal carcinoma
CPEB4: Cytoplasmic Polyadenylation Element Binding Protein 4
CPE: cytoplasmic polyadenylation element
iTRAQ: Isobaric Tags for Relative and Absolute Quantitation
IC50: half-maximal inhibitory concentration
TCGA: The Cancer Genome Atlas
RIP: RNA immunoprecipitation
IHC: Immunohistochemical
DEGs: Differentially expressed genes
DEPs: Differentially expressed proteins
EMT: epithelial-mesenchymal transition
HCC: hepatocellular carcinoma
FFEP: Formalin Fixed Paraffin Embedded
GEO: Gene Expression Omnibus
