# Supplementary material for: Molecular network analysis of CPEB4 translational control and targeting CPEB4/β-catenin to modulate invasion and migration of nasopharyngeal carcinoma cells
Source: Int J Med Sci. 2025 Feb 18;22(6):1329–43. doi: 10.7150/ijms.103024 (PMC11898850; doi:10.7150/ijms.103024)
Supplement: Supplementary file 1 — Supplementary materials and methods, figures. [file ijmsv22p1329s1.pdf]

Supplementary Figures

Fig. S1: **Correlation of CPEB4 with HNSCC Clinical Features**

(A-C) CPEB4 expression correlated significantly with N stage, M stage, and tumor grading.

(D) No significant difference in CPEB4 expression was observed between early and advanced HNSCC stages.

Fig. S2: **CPEB4 Structure and Expression in the Tumor Microenvironment**

(A) PLAAC structure analysis suggests CPEB4's potential for phase separation.

(B) Single-cell sequencing revealed that CPEB4 is highly expressed in immunophagocytes (e.g., plasma cells, mast cells, macrophages) in the tumor microenvironment.

Fig. S3: **CPEB4 Non-Classical Secretory Protein Prediction**

(A-D) Structural prediction showed no transmembrane domain or signal peptide, with a secP parity score of 0.91 (>0.6).

Fig. S4: **Quality Control for iTRAQ Data**

(A-D) Quality control data confirmed the reliability of iTRAQ analysis.

Fig. S5: **Functional Enrichment of DEPs Identified by iTRAQ**

GO and KEGG enrichment analyses highlighted pathways related to the differentially expressed proteins identified by iTRAQ.

Fig.S6: Detailed Protocols for iTRAQ and RIP assay

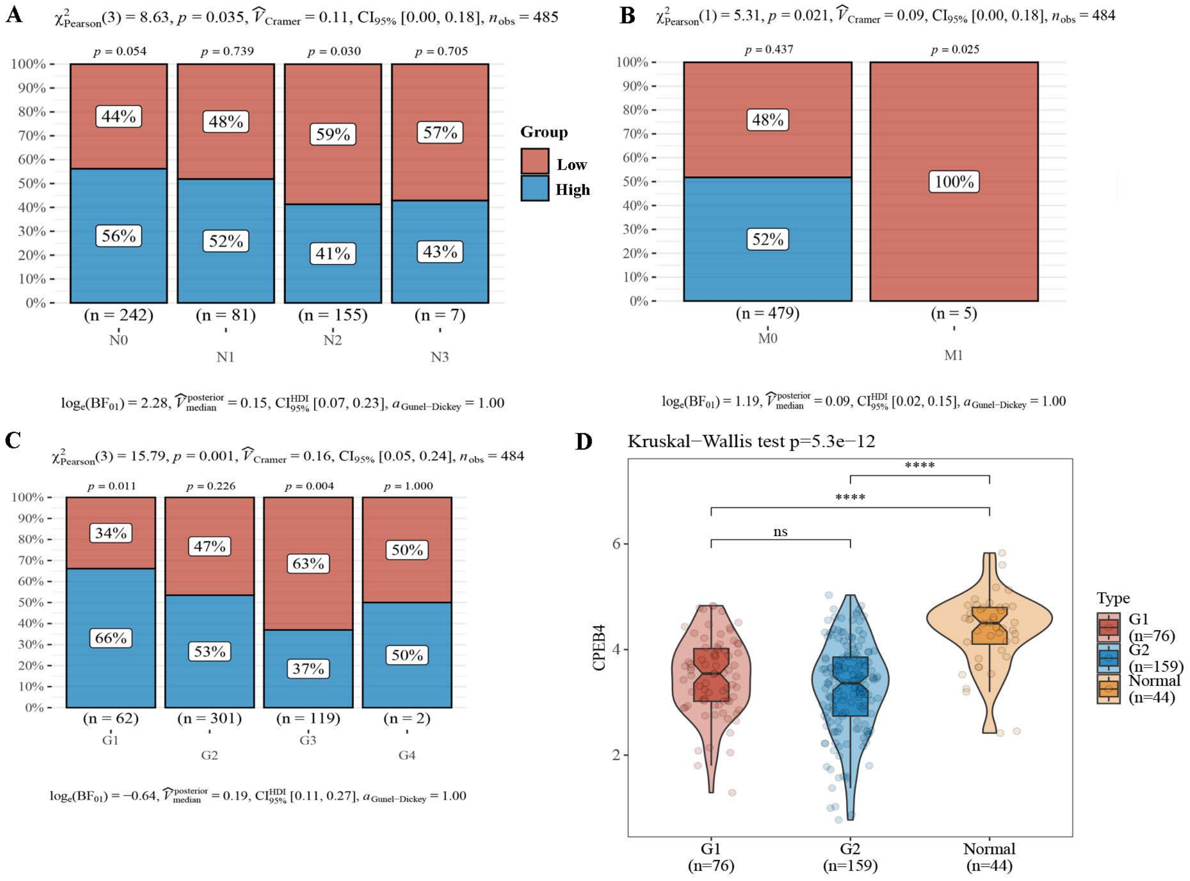

A
$\chi^2_{\text{Pearson}}(3) = 8.63, p = 0.035, \widehat{V}_{\text{Cramer}} = 0.11, \text{CI}_{95\%}$ [0.00, 0.18], $n_{\text{obs}} = 485$
p = 0.054
p = 0.739
p = 0.030
p = 0.705
44%
48%
59%
57%
56%
52%
41%
43%
(n = 242)
(n = 81)
(n = 155)
(n = 7)
N0
N1
N2
N3
Group
Low
High
$\log_e(\text{BF}_{01}) = 2.28, \widehat{V}^{\text{posterior}}_{\text{median}} = 0.15, \text{CI}^{\text{HDI}}_{95\%}$ [0.07, 0.23], $a_{\text{Gunel-Dickey}} = 1.00$
B
$\chi^2_{\text{Pearson}}(1) = 5.31, p = 0.021, \widehat{V}_{\text{Cramer}} = 0.09, \text{CI}_{95\%}$ [0.00, 0.18], $n_{\text{obs}} = 484$
p = 0.437
p = 0.025
48%
52%
100%
(n = 479)
(n = 5)
M0
M1
$\log_e(\text{BF}_{01}) = 1.19, \widehat{V}^{\text{posterior}}_{\text{median}} = 0.09, \text{CI}^{\text{HDI}}_{95\%}$ [0.02, 0.15], $a_{\text{Gunel-Dickey}} = 1.00$
C
$\chi^2_{\text{Pearson}}(3) = 15.79, p = 0.001, \widehat{V}_{\text{Cramer}} = 0.16, \text{CI}_{95\%}$ [0.05, 0.24], $n_{\text{obs}} = 484$
p = 0.011
p = 0.226
p = 0.004
p = 1.000
34%
47%
63%
50%
66%
53%
37%
50%
(n = 62)
(n = 301)
(n = 119)
(n = 2)
G1
G2
G3
G4
$\log_e(\text{BF}_{01}) = -0.64, \widehat{V}^{\text{posterior}}_{\text{median}} = 0.19, \text{CI}^{\text{HDI}}_{95\%}$ [0.11, 0.27], $a_{\text{Gunel-Dickey}} = 1.00$
D
Kruskal−Wallis test p=5.3e−12
****
****
ns
CPEB4
Type
G1 (n=76)
G2 (n=159)
Normal (n=44)

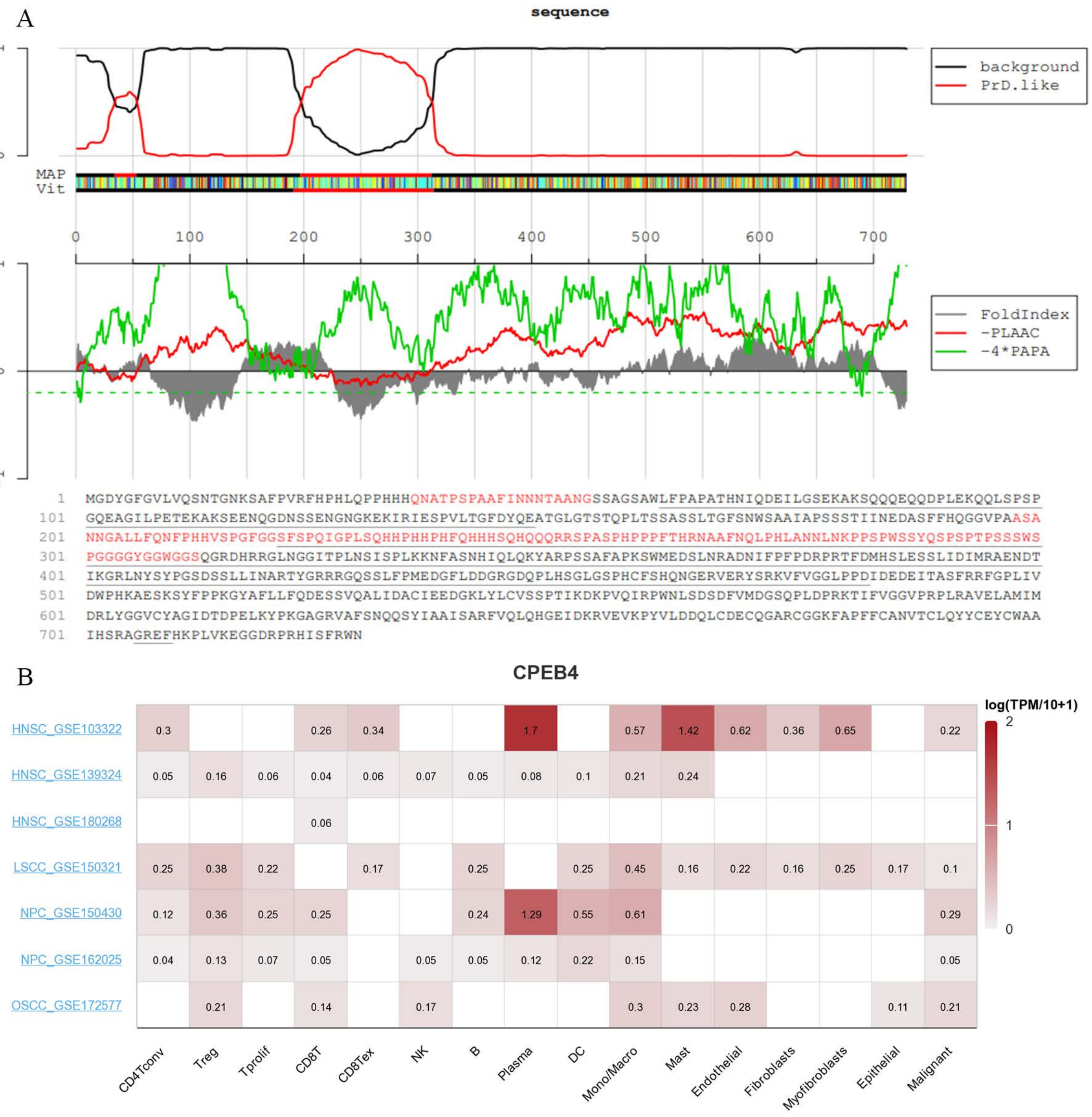
A
sequence
background
PrD.like
MAP
Vit
0
100
200
300
400
500
600
700
FoldIndex
-PLAAC
-4*PAPA
1 MGDYGFGVLVQSNTGNKSAFPVRFHPHLQPPHHHQNATPSPAAFINNNTAANGSSAGSAWLFPAPATHNIQDEILGSEKAKSQQQEQQDPLEKQQLSPSP
101 GQEAGILPETEKAKSEENQGDNSSENGNGKEKIRIESPVLTGFDYQEATGLGTSTQPLTSSASSLTGFSNWSAAIAPSSSTIINEDASFFHQGGVPAASA
201 NNGALLFQNFPHHVSPGFGGSFSPQIGPLSQHHPHHPHFQHHHSQHQQQRRSPASPHPPPFTHRNAAFNQLPHLANNLNKPPSPWSSYQSPSPTPSSSWS
301 PGGGGYGGWGGSQGRDHRRGLNGGITPLNSISPLKKNFASNHIQLQKYARPSSAFAPKSWMEDSLNRADNIFPFPDRPRTFDMHSLESSLIDIMRAENDT
401 IKGRLNYSYPGSDSSLLINARTYGRRRGQSSLFPMEDGFLDDGRGDQPLHSGLGSPHCFSHQNGERVERYSRKVFVGGLPPDIDEDEITASFRRFGPLIV
501 DWPHKAESKSYFPPKGYAFLLFQDESSVQALIDACIEEDGKLYLCVSSPTIKDKPVQIRPWNLSDSDFVMDGSQPLDPRKTIFVGGVPRPLRAVELAMIM
601 DRLYGGVCYAGIDTDPELKYPKGAGRVAFSNQQSYIAAISARFVQLQHGEIDKRVEVKPYVLDDQLCDECQGARCGGKFAPFFCANVTCLQYYCEYCWAA
701 IHSRAGREFHKPLVKEGGDRPRHISFRWN
B
CPEB4
log(TPM/10+1)
2
1
0
HNSC_GSE103322 0.3 0.26 0.34 1.7 0.57 1.42 0.62 0.36 0.65 0.22
HNSC_GSE139324 0.05 0.16 0.06 0.04 0.06 0.07 0.05 0.08 0.1 0.21 0.24
HNSC_GSE180268 0.06
LSCC_GSE150321 0.25 0.38 0.22 0.17 0.25 0.25 0.45 0.16 0.22 0.16 0.25 0.17 0.1
NPC_GSE150430 0.12 0.36 0.25 0.25 0.24 1.29 0.55 0.61 0.29
NPC_GSE162025 0.04 0.13 0.07 0.05 0.05 0.05 0.12 0.22 0.15 0.05
OSCC_GSE172577 0.21 0.14 0.17 0.3 0.23 0.28 0.11 0.21
CD4Tconv
Treg
Tprolif
CD8T
CD8Tex
NK
B
Plasma
DC
Mono/Macro
Mast
Endothelial
Fibroblasts
Myofibroblasts
Epithelial
Malignant

A

Network 1	Network 2	Network 3	SecP score	Sequence name
0.871804	0.989615	0.869438	0.910286	Sequence

B

TMHMM result

```
# WEBSEQUENCE Length: 729
# WEBSEQUENCE Number of predicted TMHs:  0
# WEBSEQUENCE Exp number of AAs in TMHs: 0.04522
# WEBSEQUENCE Exp number, first 60 AAs:  0.00018
# WEBSEQUENCE Total prob of N-in:        0.00004
WEBSEQUENCE     TMHMM2.0        outside      1    729
```

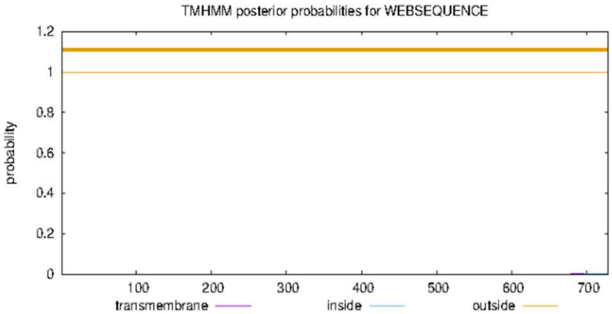

C

Sequence

Prediction: Other

Protein type	Signal Peptide (Sec/SPI)	Other
Likelihood	0.0012	0.9988

D

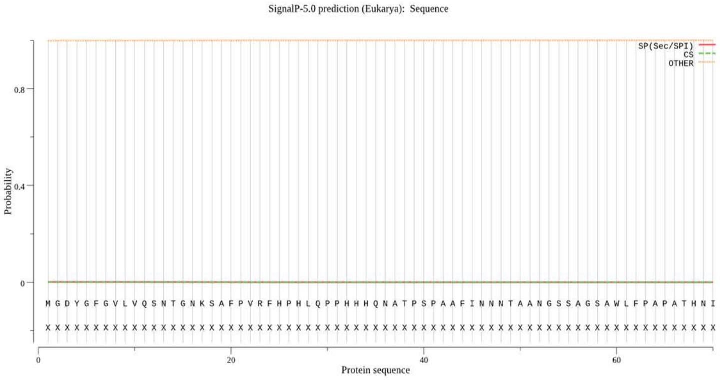

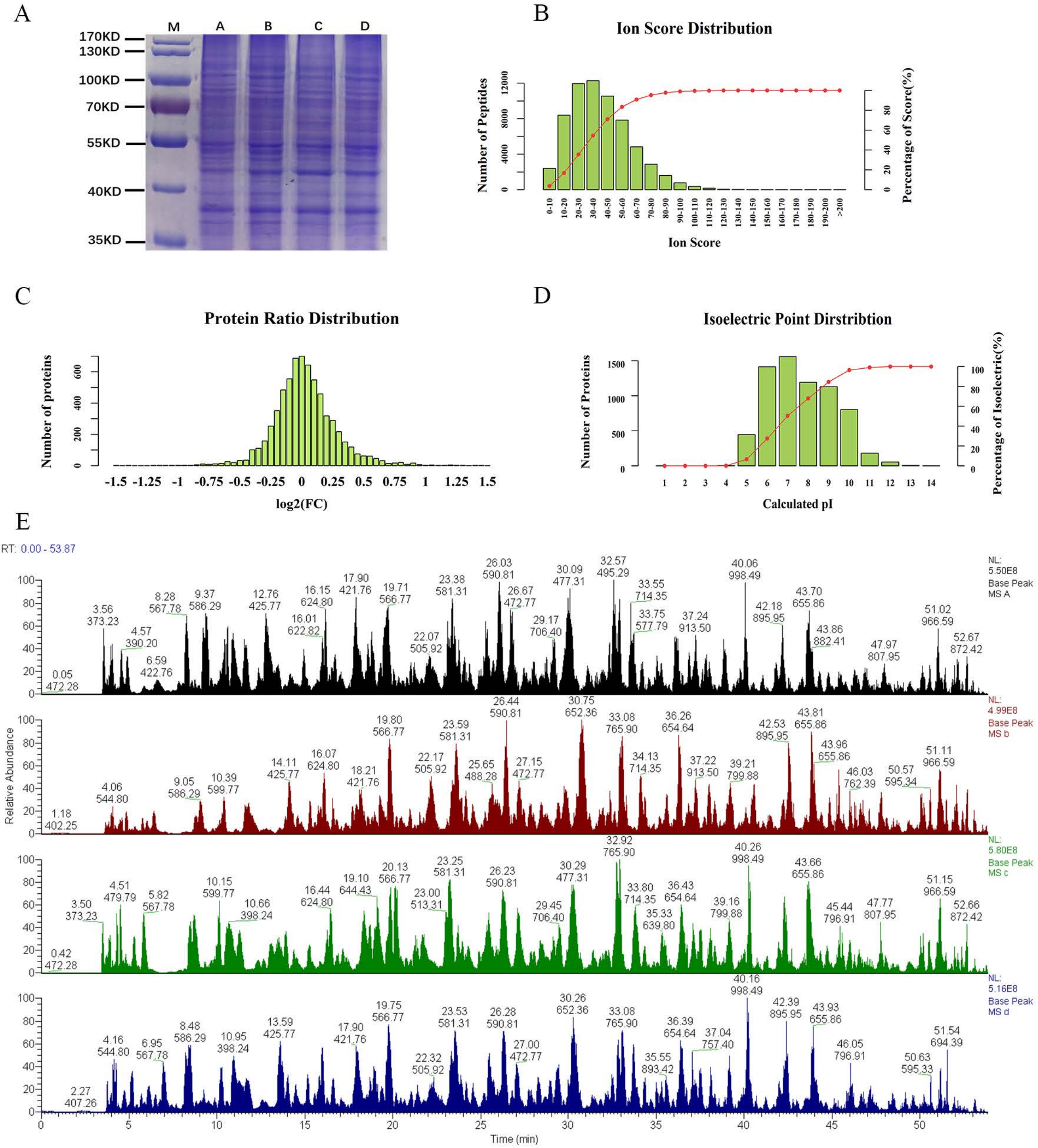

A
M
A
B
C
D
170KD
130KD
100KD
70KD
55KD
40KD
35KD
B
Ion Score Distribution
Number of Peptides
Percentage of Score(%)
Ion Score
C
Protein Ratio Distribution
Number of proteins
log2(FC)
D
Isoelectric Point Dirstribtion
Number of Proteins
Percentage of Isoelectric(%)
Calculated pI
E
RT: 0.00 - 53.87
Relative Abundance
Time (min)
NL: 5.50E8 Base Peak MS A
NL: 4.99E8 Base Peak MS b
NL: 5.80E8 Base Peak MS c
NL: 5.16E8 Base Peak MS d

GO enrichment analysis of Differently expressed proteins (DEPs) in Group A/C

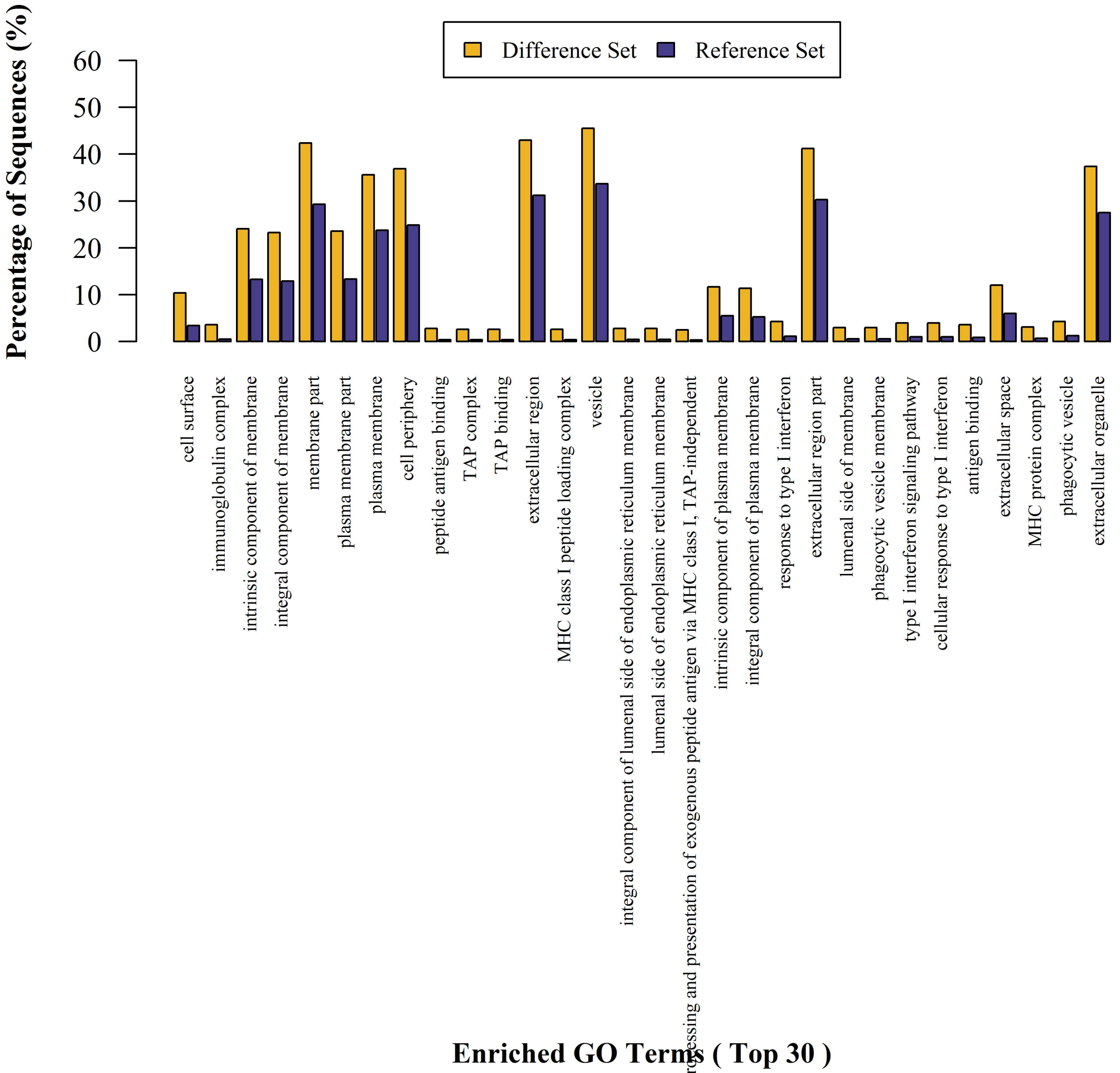

GO enrichment analysis of
Differently expressed proteins (DEPs) in Group A/C

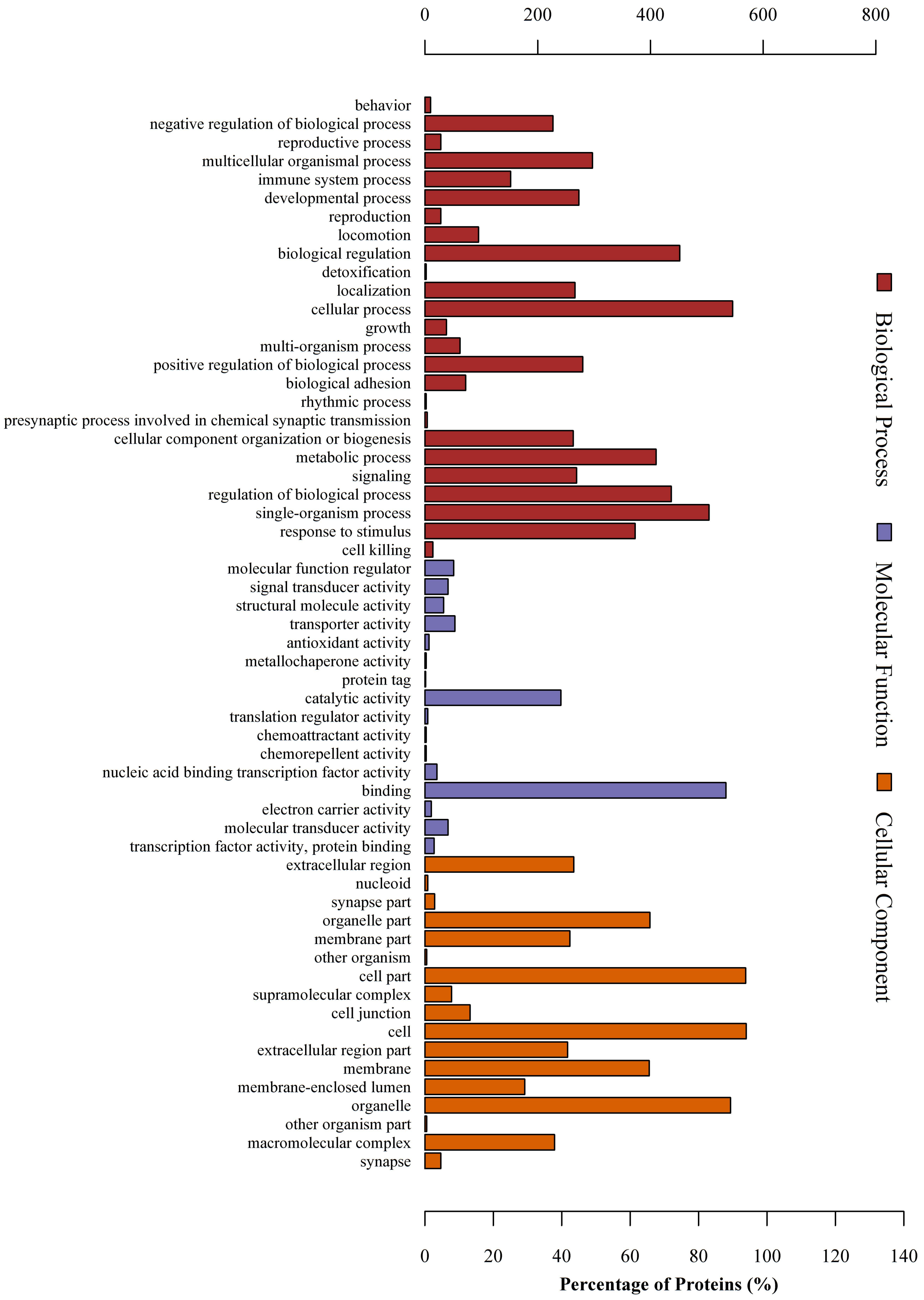

GO enrichment analysis of Differently expressed proteins (DEPs) in Group B/D

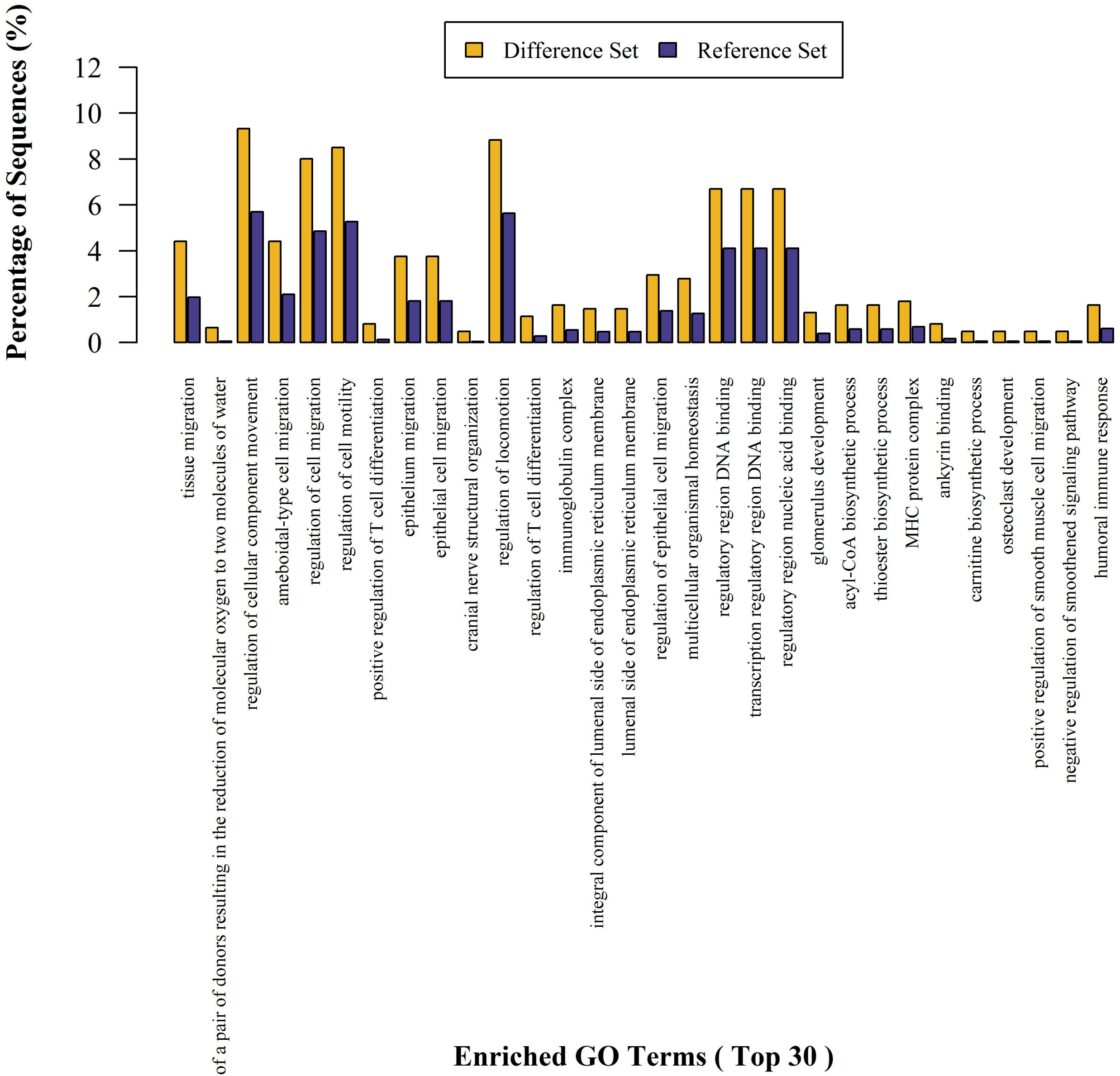

GO enrichment analysis of Differently expressed proteins (DEPs) in Group B/D

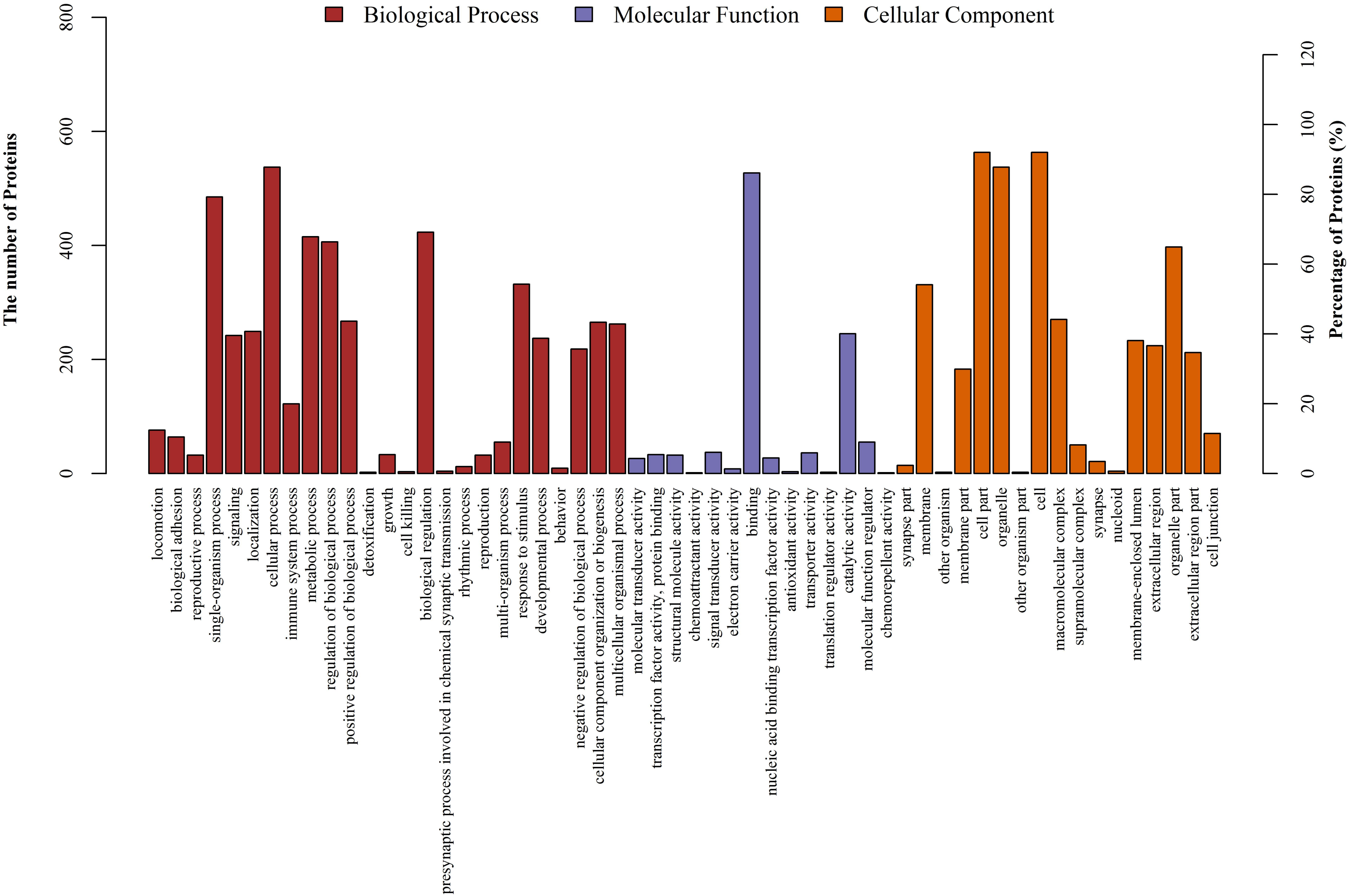

KEGG enrichment analysis of Differently expressed proteins (DEPs) in Group A/C

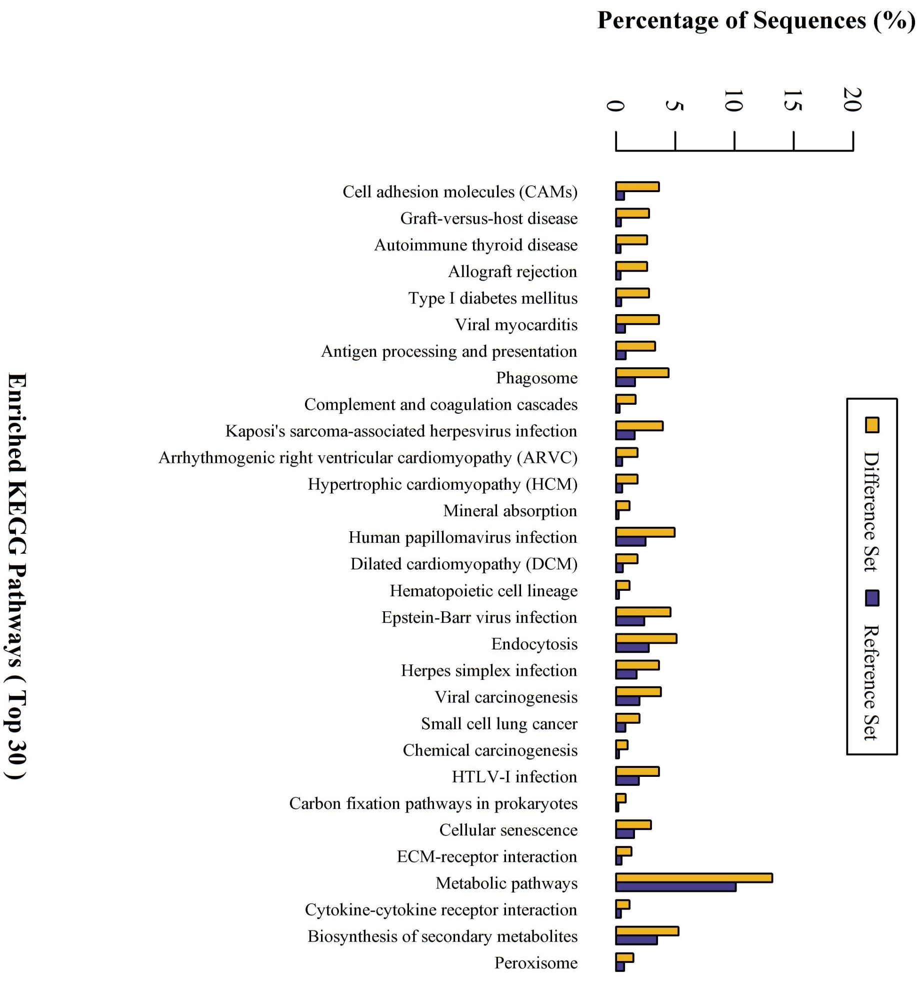

KEGG enrichment analysis of Differently expressed proteins (DEPs) in Group B/D

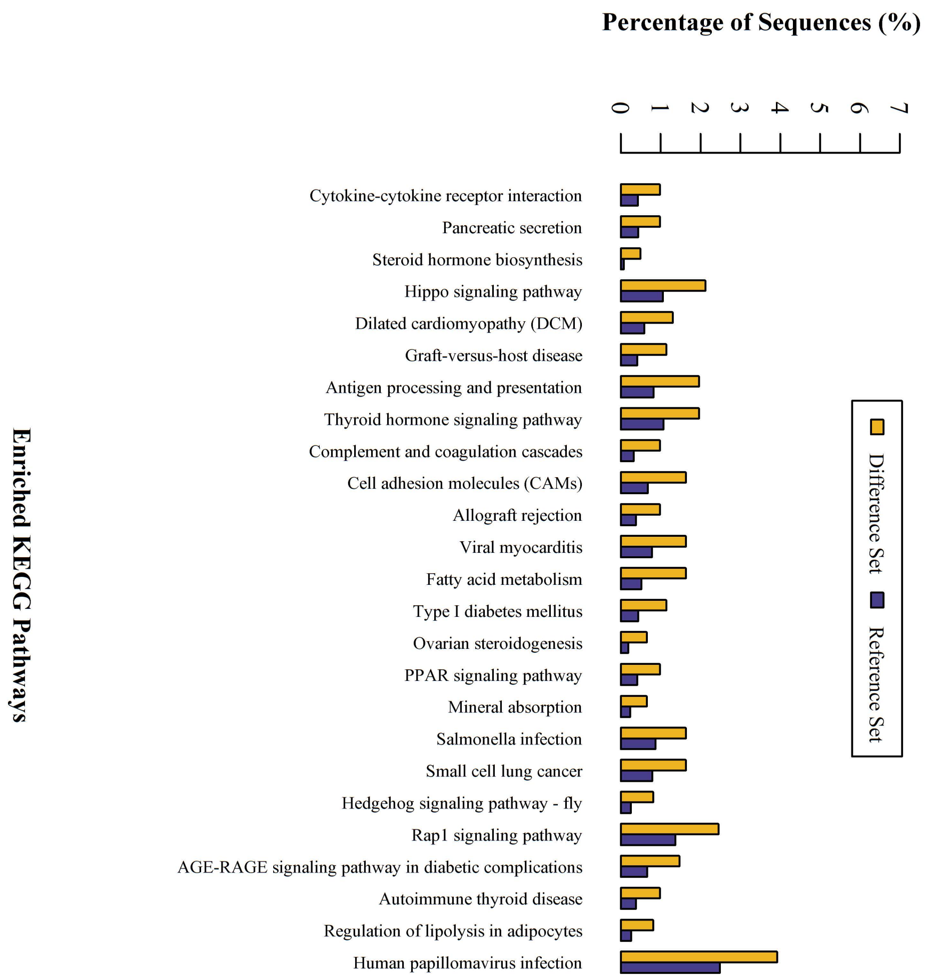

Fig.S6: Detailed Protocols for iTRAQ and RIP assay

iTRAQ Experiment Protocol

1. Sample Preparation: SDT Lysis Method
- Add an appropriate amount of SDT lysis buffer to the sample. Sonication is optional for solution-based samples.
- Heat in a boiling water bath for 15 minutes.
- Centrifuge at 14,000g for 15 minutes and collect the supernatant.
- Quantify protein concentration using the BCA method.
- Aliquot the sample and store at -80°C.

2. SDS-PAGE Electrophoresis
- Take 20 μg of protein from each sample and mix with 5X loading buffer.
- Heat in a boiling water bath for 5 minutes and perform 12% SDS-PAGE electrophoresis at a constant voltage of 250V for 40 minutes.
- Stain the gel with Coomassie Brilliant Blue.

3. FASP Digestion
- Take 30 μL of protein solution for each sample and add DTT to a final concentration of 100 mM.
- Heat in a boiling water bath for 5 minutes, then cool to room temperature.
- Add 200 μL UA buffer, mix thoroughly, and transfer to a 30 kD ultrafiltration centrifuge tube.
- Centrifuge at 14,000g for 15 minutes and discard the filtrate (repeat this step once).
- Add 100 μL IAA buffer (100 mM IAA in UA buffer), shake at 600 rpm for 1 minute, and incubate in the dark at room temperature for 30 minutes.
- Centrifuge at 14,000g for 15 minutes.
- Add 100 μL UA buffer and centrifuge at 14,000g for 15 minutes. Repeat this step twice.
- Add 100 μL of 10X diluted Dissolution buffer, centrifuge at 14,000g for 15 minutes, and repeat this step twice.
- Add 40 μL Trypsin buffer (4 μg Trypsin in 40 μL Dissolution buffer), shake at 600 rpm for 1 minute, and incubate at 37°C for 16-18 hours.
- Transfer to a new collection tube and centrifuge at 14,000g for 15 minutes.
- Add 40 μL of 10X diluted Dissolution buffer, centrifuge at 14,000g for 15 minutes, and collect the filtrate.
- Quantify peptides using a NanoDrop 2000.

4. iTRAQ Labeling
- Take 100 μg of peptides from each sample and label according to the instructions provided in the AB SCIEX iTRAQ labeling kit.

5. High pH Reversed-Phase (RP) Fractionation
- Mix the labeled peptides from each group and fractionate them using the Agilent 1260 Infinity II HPLC system.
- Buffer A: 10 mM ammonium formate, 5% ACN, pH 10.0; Buffer B: 10 mM ammonium formate, 85% ACN, pH 10.0.
- Equilibrate the column with Buffer A, then load the sample manually onto the column for

separation at a flow rate of 1 mL/min.

- Gradient:
  - ➢ 0–25 min: 0% Buffer B.
  - ➢ 25–30 min: linear gradient of Buffer B from 0% to 7%.
  - ➢ 30–65 min: linear gradient of Buffer B from 7% to 40%.
  - ➢ 65–70 min: linear gradient of Buffer B from 40% to 100%.
  - ➢ 70–85 min: maintain Buffer B at 100%.
- Monitor absorbance at 214 nm during elution and collect fractions at 1-minute intervals, resulting in approximately 36 fractions. Lyophilize the samples and redissolve them in 0.1% FA. Combine them into N fractions (refer to the experimental design for N).

6. Mass Spectrometry Analysis

Easy-nLC Chromatography:

- Separate each sample using the Easy nLC system with nano-flow rates.
- Buffer A: 0.1% formic acid in water; Buffer B: 0.1% formic acid in 80% acetonitrile.
- Equilibrate the column with 100% Buffer A. Load the sample onto the analytical column (Thermo Scientific, Acclaim PepMap RSLC 50 μm × 15 cm, Nano Viper, P/N 164943) via an auto-sampler. Flow rate: 300 nL/min.

Mass Spectrometry Identification:

- Analyze samples separated by chromatography using the Q-Exactive mass spectrometer.
- Analysis duration: 60/120 minutes (depending on the experiment).
- Detection mode: Positive ion mode.
- Precursor ion scan range: 350–1800 m/z.
- MS1 resolution: 70,000; AGC target: 3e6; Maximum IT: 50 ms.
- Collect 10 MS2 spectra (fragment ion spectra) after each full MS scan.
- MS2 parameters:
  - ➢ Activation type: HCD.
  - ➢ Isolation window: 2 m/z.
  - ➢ MS2 resolution: 17,500.
  - ➢ Microscans: 1.
  - ➢ Maximum IT: 45 ms.
  - ➢ Normalized collision energy: 30 eV.

7. Data Analysis

- Analyze raw mass spectrometry data (RAW files) using Mascot 2.5 and Proteome Discoverer 2.1 for database search and quantitative analysis.

Database Parameters:

- Protein Database: Public databases (e.g., uniprot_mouse_20141212.fasta) or project-specific custom databases.
- Enzyme: Trypsin.
- Max Missed Cleavages: 2.
- Instrument: ESI-TRAP.
- Precursor Mass Tolerance: ±20 ppm.
- Fragment Mass Tolerance: 0.1 Da.
- Use Average Precursor Mass: False.
- Modification Groups:

- iTRAQ 4/8plex (depending on the project).
- Dynamic Modifications: Oxidation (M), Acetyl (Protein N-term), Deamidated (NQ).
- Static Modifications: Carbamidomethyl (C).
- Database Pattern: Decoy (for FDR calculation).
- Peptide FDR: ≤0.01.

RNA Immunoprecipitation (RIP) assay Experiment Protocol

Kit used for RIP: Magna RIPTM RNA-Binding Protein Immunoprecipitation Kit（17-700）

(1) Preparation of Cell Lysates
1. Culture an appropriate number of CNE-2 cells in culture dishes.
2. Prepare the complete RIP lysis buffer by mixing 100 μL of RIP lysis buffer with 0.5 μL of protease inhibitor cocktail and 0.25 μL of RNase inhibitor. Mix well and keep on ice until use.

(2) Preparation of Magnetic Beads
1. Transfer 50 μL of magnetic beads and 500 μL of RIP washing buffer into each centrifuge tube. Place the tubes on a magnetic rack and discard the supernatant, leaving the beads in the tube. Add 500 μL of RIP washing buffer to each tube, then add 5 μg of the target antibody. Incubate the mixture at room temperature on a rotator for 30 minutes.
2. Perform a brief, low-speed centrifugation and place the tubes back on the magnetic rack to discard the supernatant. Repeat this washing step once. Add 500 μL of RIP washing buffer to each tube and keep on ice.

(3) Preparation of RNA-Protein Complexes
1. Prepare the RIP immunoprecipitation buffer by mixing 860 μL of RIP washing buffer, 35 μL of 0.5 M EDTA, and 5 μL of RNase inhibitor.
2. Place the magnetic bead-antibody complexes obtained in step (2) on a magnetic rack and discard the supernatant. Add 900 μL of RIP immunoprecipitation buffer, then thaw and centrifuge the lysate prepared in step (1). Transfer 100 μL of the supernatant to the tube containing the bead-antibody complexes. The final reaction volume should be 1 mL.
3. Take 10 μL of the lysate supernatant from step (1) and transfer it to a new tube as the input control. Store this tube at -80°C.
4. Incubate all tubes on a rotator at 4°C overnight. After incubation, place the tubes on a magnetic rack and discard the supernatant. Wash the beads with 500 μL of RIP washing buffer, discard the supernatant, and repeat this washing step once more.

(4) RNA Purification
1. Prepare the proteinase K buffer by mixing 117 μL of RIP washing buffer, 18 μL of proteinase K (10 mg/mL), and 15 μL of 10% SDS.
2. Resuspend the bead complexes obtained in step (3) in the prepared proteinase K buffer. Thaw the input sample on ice and add it to the proteinase K buffer. Incubate all tubes in a water bath at 55°C for 30 minutes. Place the tubes on a magnetic rack and transfer the supernatant to a new centrifuge tube. Add 250 μL of RIP washing buffer.
3. Add 400 μL of a chloroform: phenol: isoamyl alcohol mixture (125:24:1) to each tube. Vortex briefly and centrifuge at 14,000 rpm for 10 minutes at room temperature. Transfer 350 μL of the aqueous phase to a new tube and add 400 μL of chloroform. Centrifuge at 14,000

rpm for 10 minutes at room temperature. Transfer 300 μL of the aqueous phase to a new tube. Add 50 μL of Salt Solution I, 15 μL of Salt Solution II, 5 μL of glycogen, and 850 μL of absolute ethanol. Incubate overnight at -80°C to precipitate RNA. Centrifuge at 14,000 rpm for 30 minutes at 4°C and discard the supernatant.

4. Wash the RNA pellet once with 80% ethanol, centrifuge at 14,000 rpm for 15 minutes at 4°C, and discard the supernatant. Air-dry the pellet and resuspend it in 15 μL of nuclease-free water. Keep the tubes on ice until further use.

(5) RNA Quality Assessment

Assess RNA concentration and purity using a UV-visible spectrophotometer to measure absorbance.

(6) Analysis of Immunoprecipitated RNA

1. Genomic DNA Removal Reaction

Prepare a 10 μL reaction mixture on ice as follows:

- 2 μL of 5x gDNA Eraser Buffer
- 1 μL of gDNA Eraser
- RNA (≤1 μg)
- Nuclease-free water to 10 μL

Incubate the mixture in a PCR machine under the following conditions:

- ➢ 42°C for 2 minutes or at room temperature for 5 minutes.
- ➢ Hold at 4°C indefinitely.

2. Reverse Transcription Reaction

Prepare a 20 μL reaction mixture as follows:

- 10 μL of the genomic DNA removal reaction product
- 1 μL of PrimeScript RT Enzyme Mix 1
- 1 μL of RT Primer Mix
- 2 μL of 5x PrimeScript Buffer 2
- 4 μL of nuclease-free water

Perform reverse transcription in a PCR machine under the following conditions:

- ➢ 37°C for 15 minutes
- ➢ 85°C for 5 seconds
- ➢ Hold at 4°C indefinitely.

3. Amplification Reaction

Prepare a 25 μL reaction mixture on ice as follows:

- 12.5 μL of GoTaq® Green Master Mix (2x)
- 0.25-0.5 μL of upstream primer (10 μM)
- 0.25-0.5 μL of downstream primer (10 μM)
- 1-5 μL of DNA template
- Nuclease-free water to 25 μL

Mix gently and perform amplification in a PCR machine under the following conditions:

- ➢ 95°C for 3 minutes (1 cycle)
- ➢ 95°C for 30 seconds, 55°C for 30 seconds, 72°C for 1 minute (35 cycles)
- ➢ 72°C for 5 minutes
- ➢ Hold at 4°C indefinitely.

4. Agarose Gel Electrophoresis

- Prepare 1x TAE buffer by diluting 2 mL of 50x TAE to 100 mL with water.
- Melt 1.5 g of agarose in 100 mL of 1x TAE buffer, then cool to 50-60°C and add ethidium bromide (final concentration: 0.5 µg/mL). Pour the gel into a tray, avoiding bubbles, and let it solidify.
- Load 5 µL of a 50 bp DNA ladder and 20 µL of PCR products into the wells.
- Run the gel at 80 V and observe under UV light at 254 nm. Record images using gel documentation software.
